# PIEZO1 gain-of-function mutation drives cardiomyopathy by disrupting myocardial lipid homeostasis besides iron overload

**DOI:** 10.1126/sciadv.ady9242

**Published:** 2025-11-14

**Authors:** Cuiqin Fan, Han Du, Song Sun, Huixia Lu, Yanming Wang, Fujian Lu, Qian Wang, Cheng Zhang, Li Xue, Chang Pan, Hongwei Yue, Hao Wang, Juying Qian, Sumei Cui, Yuguo Chen, Feng Xu

**Affiliations:** ^1^ Department of Emergency Medicine, Qilu Hospital of Shandong University, Jinan, China.; ^2^ Shandong Provincial Clinical Research Center for Emergency and Critical Care Medicine, Institute of Emergency and Critical Care Medicine of Shandong University, Chest Pain Center, Qilu Hospital of Shandong University, Jinan, China.; ^3^ Medical and Pharmaceutical Basic Research Innovation Center of Emergency and Critical Care Medicine, China’s Ministry of Education, Shandong Provincial Engineering Laboratory for Emergency and Critical Care Medicine, Key Laboratory of Emergency and Critical Care Medicine of Shandong Province, Key Laboratory of Cardiopulmonary-Cerebral Resuscitation Research of Shandong Province, Qilu Hospital of Shandong University, Jinan, China.; ^4^ NMPA Key Laboratory for Clinical Research and Evaluation of Innovative Drug, Qilu Hospital of Shandong University, Jinan, China.; ^5^ State Key Laboratory for Innovation and Transformation of Luobing Theory, Key Laboratory of Cardiovascular Remodeling and Function Research, Chinese Ministry of Education, Chinese National Health Commission and Chinese Academy of Medical Sciences, Qilu Hospital of Shandong University, Jinan, China.; ^6^ Department of Cardiology, Qilu Hospital of Shandong University, Jinan, China.; ^7^ Institutes of Biomedical Sciences, Department of Cardiology, Zhongshan Hospital, Shanghai Institute of Cardiovascular Diseases, Fudan University, Shanghai, China.; ^8^ Department of Radiology, Qilu Hospital of Shandong University, Jinan, China.; ^9^ Department of Critical Care Medicine, Qilu Hospital of Shandong University, Jinan, China.; ^10^ Department of Cardiology, Zhongshan Hospital, Shanghai Institute of Cardiovascular Disease, Fudan University, Shanghai, China.

## Abstract

As a mechanosensitive channel, PIEZO1 translates mechanical stretching of cardiomyocytes into Ca^2+^ signaling, underpinning the Frank-Starling law. This mechanism contributes to compensatory responses in heart failure. However, the relationship between PIEZO1 mutations and the development of cardiomyopathy remains unclear. Acute heart failure complicated with severe myocardial iron deposition was identified in the 31-year-old male proband of PIEZO1^D669Y^ variant. However, PIEZO1 gain-of-function (GOF) mutation D674Y mice and cardiomyocyte-specific *Piezo1* overexpression disrupted cardiac function besides iron overload. Using single-cell RNA sequencing, we observed suppression of lipid metabolism pathways in cardiomyocytes with the PIEZO1 GOF mutation, with forkhead box O3 (FOXO3) as a key mediator in lipid metabolism pathways. Specifically, the PIEZO1 GOF mutation increased Ca^2+^ levels, leading to calcium- and calmodulin-dependent protein kinase II (CaMKII) activation and subsequent FOXO3 down-regulation. Together, we demonstrate that PIEZO1 GOF mutation contributes to cardiomyopathy by disrupting myocardial lipid metabolism. This study challenges the current clinical focus on iron-related mechanisms in cardiomyopathy and supports PIEZO1 as a potential candidate for future genetic screening for cardiomyopathy.

## Teaser

PIEZO1 GOF mutation alters cardiac function via disrupted calcium signaling and lipid metabolism in cardiomyocytes.

## INTRODUCTION

Cardiomyopathies are the second leading cause of sudden cardiac death and a major contributor to heart failure worldwide (*1*). Among these, dilated cardiomyopathy (DCM) is the most prevalent (1 in 250 individuals), exceeding hypertrophic cardiomyopathy (1 in 500) in frequency, with 5 to 15% of patients harboring a likely pathogenic variant (*2*). Advances in genetic testing over the past decade have facilitated the identification of numerous gene variants associated with this condition (*3*). To interpret these genetic findings accurately and determine the specific contributions of gene variants to DCM pathogenesis, comprehensive investigations in both clinical and experimental research settings are essential.

The mechanosensitive ion channel PIEZO1 plays a critical role in pathophysiological changes in the heart, with low baseline expression in cardiomyocytes that is markedly up-regulated in DCM (*4*–*6*). Upon activation, PIEZO1 transduces mechanical load into Ca^2+^ and reactive oxygen species (ROS) signaling in cardiomyocytes, which are fundamental for regulating the heart’s mechanical activity. Elevated levels of the PIEZO1 protein have been observed in both humans and mice with heart failure (*7*). Moreover, supraphysiological overexpression of *Piezo1* in cardiomyocytes induces a DCM phenotype in mice. In addition, both the absence of *Piezo1* or its overexpression, as well as the *Piezo1* M2241R mutation, can lead to a spectrum of cardiac abnormalities ranging from hypertrophy and fibrosis to cavity enlargement and DCM (*8*). These findings suggest a broader role for PIEZO1 dysfunction in the progression and development of DCM.

In human populations, gain-of-function (GOF) mutations in *PIEZO1* represent the most common genetic cause of hereditary xerocytosis (HX), a rare disorder frequently complicated by iron overload and compensatory anemia (*9*). Of note, an increasing number of case reports, including ours, have highlighted cardiac involvement in patients with HX, particularly the development of DCM (*10*, *11*). Specifically, both the probands and their father carrying the PIEZO1 M2007L variant exhibited clear DCM phenotypes (*10*). In addition, a small case series reported a 25-year-old patient with HX harboring the PIEZO1 V2006D variant who experienced sudden death, presumably due to myocardial iron overload leading to heart failure (*11*). Furthermore, we reported a 31-year-old male carrying the PIEZO1^D669Y^ variant who similarly presented with DCM. However, the mechanism by which GOF PIEZO1 mutations contribute to heart failure remains unclear. Given the frequent observation of myocardial iron accumulation in these patients, it is clinically assumed that iron overload is the etiology underlying PIEZO1-related cardiomyopathy (*11*–*13*). Nevertheless, this assumption remains untested. This knowledge gap limits our understanding of the underlying causes of heart failure in these patients, as well as our ability to predict patient prognosis and develop effective therapeutic interventions.

In this study, we aimed to determine the mechanisms of heart failure in carriers of GOF PIEZO1 variants by integrating data from both human and animal studies. Our results indicate that PIEZO1 GOF contributes to cardiomyopathy partly independent of iron overload. Mechanistic investigations revealed that PIEZO1 GOF increases leads to cardiolipotoxicity through calcium signal–induced activation of the calcium- and calmodulin-dependent protein kinase II (CaMKII)–forkhead box O3 (FOXO3) axis. We revealed previously unrecognized mechanisms by which PIEZO1 GOF mutations contribute to DCM patients. These findings provide crucial insights for molecular diagnosis, prognosis prediction, and the development of potential therapeutic strategies for managing PIEZO1-associated cardiomyopathy.

## RESULTS

### Clinical follow-up of a PIEZO1^D669Y^ carrier reveals discordance between cardiac iron overload and function

**Fig. 1.**
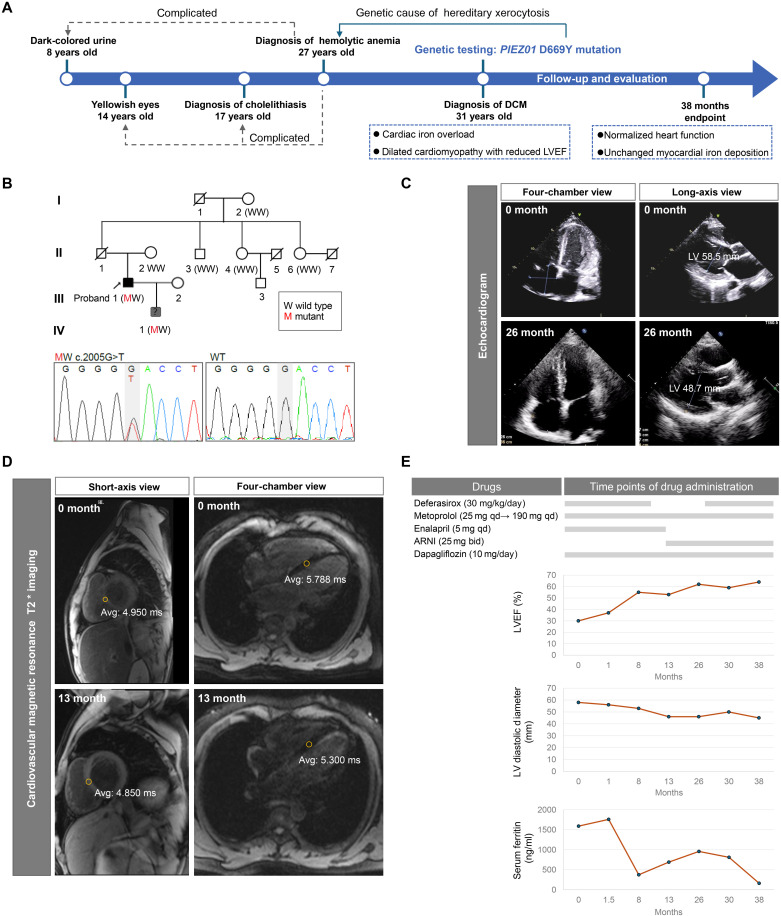
Identification of *PIEZO1*^D669Y^ variant carriers with DCM and iron overload. (**A**) Timeline depicting the proband’s (III-1) medical history, key diagnoses, and etiological analysis. (**B**) Pedigrees and Sanger validation of the PIEZO1^D669Y^ variants of the patients in the family. Upper section (pedigree chart): adapted from (*14*), with permission. (**C**) Representative four-chamber and long-axis view echocardiographic images of the proband patient (III-1) at two time points. (**D**) Cardiac MRI in four-chamber and short-axis views of the patient (III-1). Upper section (0 month): reprinted from (*14*), with permission. (**E**), The duration of medication administration (indicated by gray bars) for the proband patient (III-1) and dynamic changes in LVEF, left ventricular diameter at end-diastole (LV diastolic diameter), and serum ferritin levels over time are shown. Medications, including deferasirox (30 mg/kg/day) for iron overload, metoprolol (starting from 23.75 mg, titrated based on heart rate, reaching 190 mg by month 30 with heart rate maintained between 55 and 60 bpm), enalapril [5 mg daily (qd)], and, later, Angiotensin receptor-neprilysin inhibitor (ARNI) [25 mg twice daily (bid)] for heart failure management, and dapagliflozin (10 mg/day) as standard heart failure treatment. Avg, average.

A family with two PIEZO1^D669Y^ mutation carriers was analyzed. The proband (III-1) with normal body mass index (20.07) was admitted with acute decompensated heart failure complicated by HX, cholelithiasis, iron overload, diabetes mellitus, hypogonadism, and DCM (Fig. 1A) (*14*). The proband also presented with normal glycated hemoglobin and normal lipid profile levels (table S2). Genome exon sequencing revealed a monoallelic variant of *PIEZO1* (c.2005G > T, NM_001142864, p.D669Y) in the proband as a pathogenic gene for HX, with no known pathogenic or likely pathogenic cardiomyopathy loci detected (Fig. 1B). This variant is relatively conserved among mammals (fig. S1A). Echocardiographic assessments revealed compromised cardiac pump function, as evidenced by a diminished left ventricular ejection fraction (LVEF) (30%), along with markedly dilated cardiac chambers (LV end-diastolic diameter: 58.5 mm) and thinned ventricular walls (interventricular septum: 10 mm, LV posterior wall: 11 mm) (Fig. 1, C and E, and table S1), which are characteristic of DCM. In addition, the proband (III-1) exhibited elevated serum ferritin levels of 1590 ng/ml, exceeding the normal range of 13 to 400 ng/ml, along with a transferrin saturation of 97.6%, which was markedly greater than the normal range of 20 to 50% (table S2). The serum iron level was also increased to 45.4 μM (normal range: 9 to 27 μM) (table S2). Notably, cardiac magnetic resonance imaging (MRI) revealed a T2* relaxation time of less than 6.0 ms (normal range: > 20 ms) (*15*), indicating severe myocardial iron deposition (top panel of Fig. 1D). Given the role of PIEZO1 GOF in iron overload through the down-regulation of hepcidin (*16*) and the evidence of cardiac iron deposition via MRI, DCM secondary to iron overload resulting from PIEZO1 mutation could be one of several diagnoses.

However, iron overload typically presents with ventricular hypertrophy and preserved LVEF (*17*), rather than the normal wall thickness and reduced LVEF observed in this patient. Hence, it is speculated that other mechanisms contributed to his cardiac phenotype. Upon diagnosis, the PIEZO1^D669Y^ carrier was treated with the iron chelator deferasirox and anti–heart failure therapy including metoprolol, enalapril, and dapagliflozin (Fig. 1E). Spironolactone was not initiated due to preexisting hyperkalemia, which is also a manifestation of PIEZO1 GOF mutations. As anticipated, deferasirox effectively reduced the serum ferritin level (Fig. 1E), a marker of systemic iron burden. However, cardiac MRI revealed no obvious difference in myocardial iron overload before and after treatment (Fig. 1D, bottom panel). Despite the initial anticipation of a poor prognosis due to severe left ventricular dysfunction and acute decompensation, both the reduced LVEF and chamber enlargement gradually normalized during treatment (Fig. 1, C and E), inconsistent with the typical features of iron overload cardiomyopathy. Specially, despite fluctuations in serum ferritin upon deferasirox cessation between months 8 to 26 of therapy, improvements in LVEF and reductions in cardiac chamber dimensions continued (Fig. 1, C and E). The poor association between myocardial iron deposition and cardiac function, coupled with the occurrence of reversible heart failure that was distinct from typical iron overload cardiomyopathy, suggests that the patient’s heart failure could not be attributed only to iron overload. The proband’s 7-year-old male offspring (IV-1), a heterozygous carrier of the variant, presented with paroxysmal atrial tachycardia but no overt signs of heart failure (tables S3 and S4), which partly aligns with the age-dependent penetrance of monogenic DCM.

### Mice harboring Piezo1^D674Y^ develop DCM

To investigate the mechanisms of *PIEZO1*^D669Y^-related cardiomyopathy, we used CRISPR-Cas9 technology to generate mice with the murine Piezo1^D674Y^ mutation (fig. S2, A to C), which corresponds to the PIEZO1^D669Y^ mutation in humans. We obtained 158 wild-type (WT) mice and 60 heterozygous mutant mice (referred to as MW mice in the figures); no homozygous mutant mice were generated. The absence of homozygous mutants suggested that the homozygous mutation resulted in embryonic lethality. As a result, all phenotypic analyses were conducted on heterozygous mutants, with males and females analyzed separately to assess potential sex-specific difference.

**Fig. 2.**
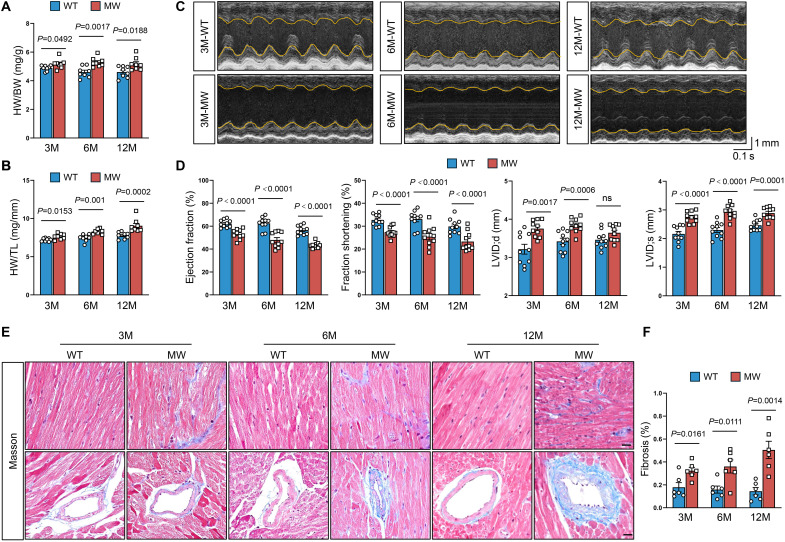
Piezo1^D674Y^ mutation impairs heart function. (**A**) HW/BW ratio of male WT and *Piezo1*^D674Y^ (MW) mice at 3 to 12 months (*n* = 8). (**B**) HW/TL ratio of male WT and MW mice at 3 to 12 months (*n* = 8). (**C**) Representative M-mode echocardiographic images of male WT and MW mice at 3 to 12 months. (**D**) Echocardiographic analysis of the ejection fraction, FS, left ventricular internal dimension at diastole (LVID;d) and left ventricular internal dimension at systole (LVID;s) of male WT and MW mice at 3 to 12 months (*n* = 10). (**E** and **F**) Representative images (E) and quantification (F) of Masson’s trichrome staining of cardiac tissues from male WT and MW mice at 3 to 12 months (*n* = 6). Scale bars, 20 μm. Two-tailed nonparametric Mann-Whitney test (B) or unpaired Student’s *t* test [(A), (B), (D), and (F)] was used. The number of samples in each group is indicated by *n*. The data are presented as the means ± SEM. M, months; ns, not significant.

In males, the mutant mice showed no notable difference in survival rate from male WT mice (fig. S2D) and exhibited no observable differences in external appearance at 3, 6, or 12 months of age (fig. S2E). However, as early as 3 months of age, the heterozygous male mice presented significantly enlarged hearts (fig. S2F), along with a significant increase in the heart weight–to–body weight (HW/BW) ratio (Fig. 2A and fig. S2G) and the heart weight–to–tibia length (HW/TL) ratio (Fig. 2B). Starting at 3 months of age, male MW mice presented significant impairment of cardiac pump function, as evidenced by a reduced LVEF and fractional shortening (FS), along with left ventricular chamber dilation (increased internal diameter and volume), but no change in ventricular wall thickness (Fig. 2, C and D; fig. S2H; and table S5). Histologically, mutant male mice presented increased cardiac fibrosis, as evidenced by increased accumulation of collagen fibers in the interstitial spaces between cardiomyocytes (Fig. 2, E and F).

Moreover, female heterozygous Piezo1^D674Y^ mice presented a similar but more delayed phenotype. At 3 months of age, female MW mice were indistinguishable from WT controls in terms of cardiac size, structure, and function (fig. S3 and table S6). However, by 6 months of age, signs of cardiac dysfunction began to emerge, including increased LV chamber dimensions, decreased LVEF and FS, and elevated HW/BW and HW/TL ratios (fig. S3, A to H, and table S6). Histological analysis at 6 months also revealed accumulation of interstitial collagen fibers (fig. S3, I and J), suggesting the initiation of pathological remodeling. These changes progressed further with age, suggesting a time-dependent decline in cardiac function and a delayed disease manifestation in female mice. Consequently, all subsequent experiments were conducted using male mice.

### Piezo1^D674Y^ mutation increases Piezo1 channel activity

**Fig. 3.**
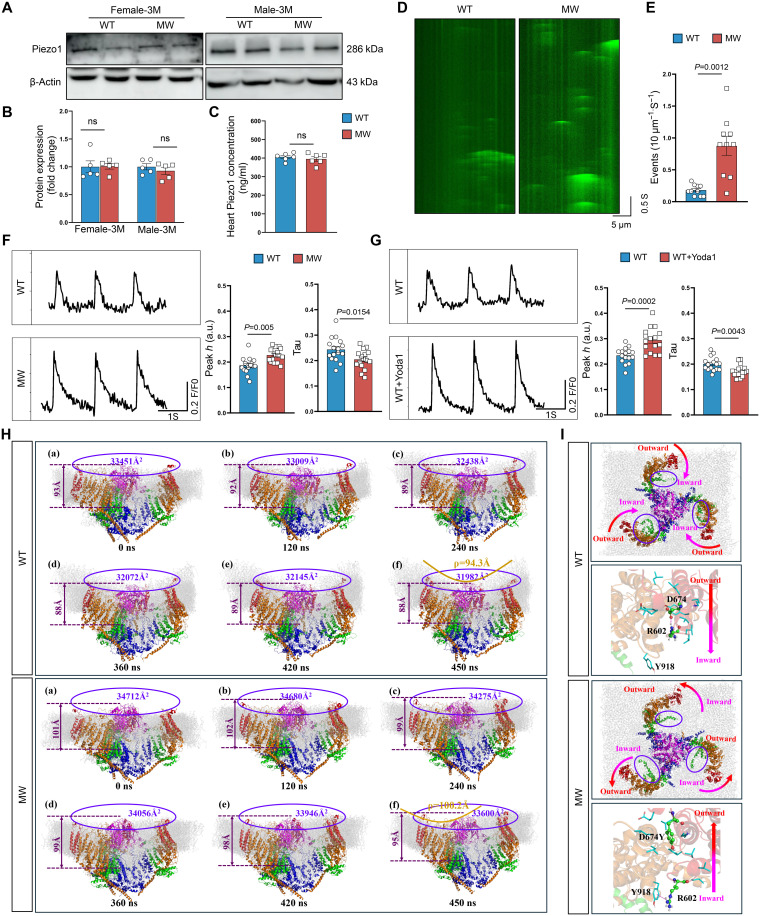
Piezo1^D674Y^ is a GOF mutation. (**A** and **B**) Representative Western blot images (A) and quantitative analyses (B) of Piezo1 in cardiac tissues from female and male mice at 3 months (*n* = 5). (**C**) Piezo1 concentration in the hearts of male mice at 3 months (*n* = 6). (**D** and **E**) Representative fluorescence surface plot (D) and quantification (E) of Ca^2+^ sparks in the indicated cardiomyocytes from male WT and MW mice (*n* = 10 cells). (**F**) Representative images and quantifications of peak *h* and Tau of cardiomyocytes Ca^2+^ transient traces recorded from male WT and MW mice (*n* = 16 cells). (**G**) Representative images and quantifications of peak *h* and Tau of cardiomyocytes Ca^2+^ transient traces recorded from male WT mice with or without Yoda1 stimulation (*n* = 16 cells). (**H**) Snapshots of the WT and MW trimers embedded in the phospholipid membrane at (a) 0 ns, (b) 120 ns, (c) 240 ns, (d) 360 ns, (e) 420 ns, and (f) 450 ns during the MD simulations. (**I**) Global view and close-up view of the structures of the WT and MW trimers after 450-ns MD simulations. Two-tailed unpaired Student’s *t* test was used [(B), (C), and (E) to (G)]. The number of samples in each group is indicated by *n*. The data are presented as the means ± SEM. a.u., arbitrary unit.

We next investigated the influence of the Piezo1^D674Y^ mutation on *Piezo1* channel. Western blot analysis of male mice heart tissues revealed that the Piezo1^D674Y^ mutation did not alter the protein level of Piezo1 (Fig. 3, A and B), which was consistent with the results of enzyme-linked immunosorbent assay (ELISA) (Fig. 3C). The mutant male cardiomyocytes exhibited an elevated Ca^2+^ spark frequency and Ca^2+^ transient amplitude (Fig. 3, D to F), probably due to increased Piezo1 channel activity. Consistently, activation of Piezo1 by Yoda1 exhibited an obvious elevation of Ca^2+^ transient amplitude and decay of Tau (Fig. 3G). Molecular dynamics (MD) simulations were subsequently used to analyze the structure of Piezo1. According to the root mean square deviation, the WT Piezo1 trimer and the Piezo1^D674Y^ trimer fully relaxed and became equalized in the last 90- and 150-ns MD simulations, respectively (fig. S4A). During the equilibrium stages of the 450-ns MD simulation, we analyzed the fluctuation value (root mean square fluctuation/B factor ratio) of each residue in the WT and mutant trimers. The Piezo1^D674Y^ trimer showed greater flexibility than the WT trimer (fig. S4B), indicating slower channel kinetics, which likely prolonged the opening time of the Piezo1 channel (*18*). Structural snapshots from the simulations demonstrated that the Piezo1^D674Y^ mutant was more inclined to adopt a flattened state, as evidenced by the fact that the vertical and projected areas of the mutant were higher than those of the WT (Fig. 3H and movies S1 and S2), as larger vertical and projected areas are typically associated with activated ion channels (*19*). The inward movement of the D674 residue in WT Piezo1 allows it to form a salt bridge with R602, but this movement is abolished in the mutant, causing a cation-π interaction to form between R602 and Y918 and Y674 to adopt an outward orientation (Fig. 3I), potentially influencing the movement of the blade region of the Piezo1 trimer. Superimposition of the structures indicated that in the mutant, the propeller blades tended to flip outward and downward (fig. S4C), causing the Piezo1^D674Y^ mutant to have a larger radius of gyration (fig. S4D). The structural changes near this mutation site ultimately led to changes in the structure of the ion channel. In the WT system, the ion transport channel was elongated, whereas in the mutant system, this channel had a “short and fat” shape, which theoretically increased the efficiency of ion transport through the channel (fig. S4, E and F). We then analyzed Ca^2+^ ion permeation in the WT and mutant systems. As expected, in the WT system, the transit of Ca^2+^ ions through the ion channel were noticeably hindered over the first 22 ns, especially between 6 and 20 ns, whereas in the mutant system, the wider ion channel allowed faster permeation of Ca^2+^ ions, with only minor obstruction between 8 and 12 ns, resulting in a quicker transit time of 16 ns (fig. S4G). Our MD simulations suggest that the D674Y mutation might lower the threshold for opening or stabilizing an expanded (pre-open) conformation of the Piezo1 channel, even in the absence of mechanical load, allowing for faster calcium permeation. These analyses strongly suggest that the Piezo1^D674Y^ mutation acts as a GOF mutation that promotes Piezo1 activation.

### Pharmacological modulation of PIEZO1 channel activity influences cardiac function and remodeling

We subsequently examined whether Piezo1 channel inhibition could rescue heart dysfunction in male Piezo1^D674Y^ mice. As expected, treatment of Piezo1^D674Y^ mice at 8-week-old with Grammostola spatulata mechanotoxin 4 (GsMTx4), an inhibitor of Piezo1 (*20*), successfully normalized the LVEF and FS (fig. S5, A and B, and table S7) and prevented interstitial fibrosis by 12 weeks of age (fig. S5, C and D). The relatively modest effect of GsMTx4 on left ventricular internal dimension (LVID), compared to that of the gene mutation, likely reflects the limited treatment duration, which may not suffice to induce structural reversal.

Next, we administered Yoda1, a chemical activator of the Piezo1 channel (*21*), to WT mice at 8 weeks old. We found a significant decrease in the LVEF and FS (fig. S6, A and B, and table S8) and increased fibrosis by 12 weeks of age (fig. S6, C and D), consistent with the cardiac phenotype observed in Piezo1^D674Y^ mice. Collectively, these results suggest that Piezo1 channel activity has direct effects on cardiac function.

### PIEZO1 GOF impairs cardiac function and induces remodeling before evident cardiac iron overload

**Fig. 4.**
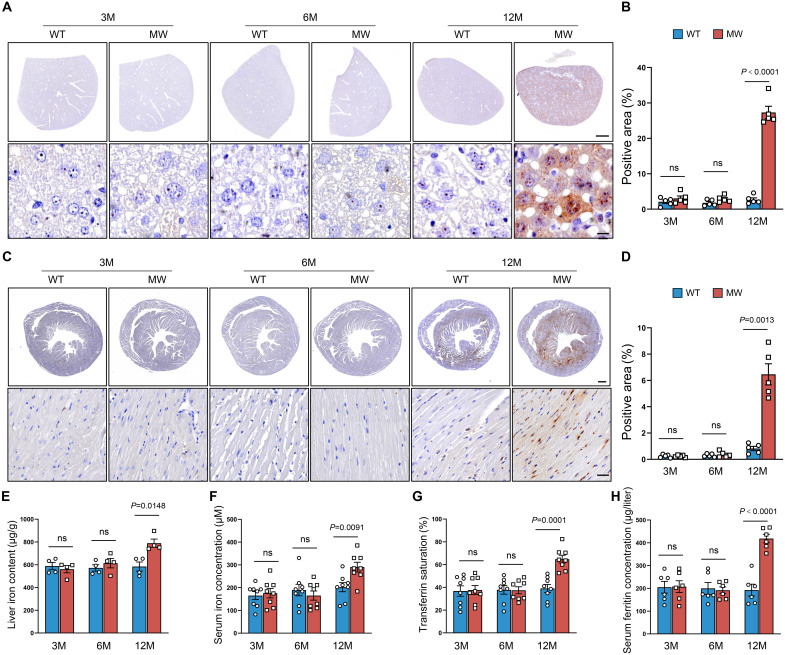
Piezo1 GOF causes cardiac dysfunction without iron overload. (**A** and **B**) Representative images (A) and quantitative (B) of Perls Prussian blue staining of liver tissues from male WT and MW mice at 3 to 12 months (*n* = 5). Scale bars, 1 mm and 10 μm. (**C** and **D**) Representative images (C) and quantitative (D) of Perls Prussian blue staining of cardiac tissues from male WT and MW mice at 3 to 12 months (*n* = 5). Scale bars, 1 mm and 10 μm. (**E**) Iron content in the livers from male WT and MW mice (*n* = 4). (**F**) Iron content in the serum of male WT and MW mice (*n* = 8). (**G**) Transferrin saturation in the serum of male WT and MW mice (*n* = 8). (**H**) Ferritin concentrations in the serum of male WT and MW mice (*n* = 6). Two-tailed unpaired Student’s *t* test was used [(B) and (D) to (H)]. The number of samples in each group is indicated by *n*. The data are presented as the means ± SEM.

We next examined the relationship between iron levels and heart function in male mice. To assess iron deposition, we performed Perls Prussian blue staining, starting with the liver due to its high sensitivity to iron (*22*). The mutant mice showed no significant iron deposition in hepatocytes (Fig. 4, A and B) or cardiomyocytes until 12 months of age (Fig. 4, C and D), which was further supported by ELISA (Fig. 4E). Serum analysis revealed that iron concentration, transferrin saturation, and ferritin levels were significantly elevated in Piezo1^D674Y^ mice compared with WT mice only at 12 months (Fig. 4, F to H), with no differences detected at 3 or 6 months. At the transcript level, the levels of genes in the hepcidin-ferroportin regulatory axis, including transferrin receptor 1 (TfR1), ferritin heavy chain (FtH), ferritin light chain (FtL), and ferroportin (Fpn), in cardiac tissues were similar between 3-month-old Piezo1^D674Y^ and WT mice (fig. S7A). Protein analyses revealed no differences in ferritin, ferroportin (FPN), solute carrier family 7 member 11 (SLC7A11), or glutathione peroxidase 4 (GPX4) expression between the groups at 3 months of age (fig. S7, B and C). Given that even trace amounts of iron in cardiomyocytes can induce oxidative damage (*23*), we examined the levels of markers associated with ferroptosis and oxidative stress. There were no notable differences in the transcript levels of ferroptosis markers, such as superoxide dismutase 2 (SOD2) and prostaglandin-endoperoxide synthase 2 (PTGS2) (fig. S7D). These findings suggest that the Piezo1^D674Y^ mutation results in iron overload by 12 months of age. Nevertheless, heart function impairment was evident in 3-month-old mice, suggesting that factors other than iron overload contribute to heart failure.

### Cardiomyocyte lipid metabolism imbalance in the hearts of mice with Piezo1 GOF mutation

**Fig. 5.**
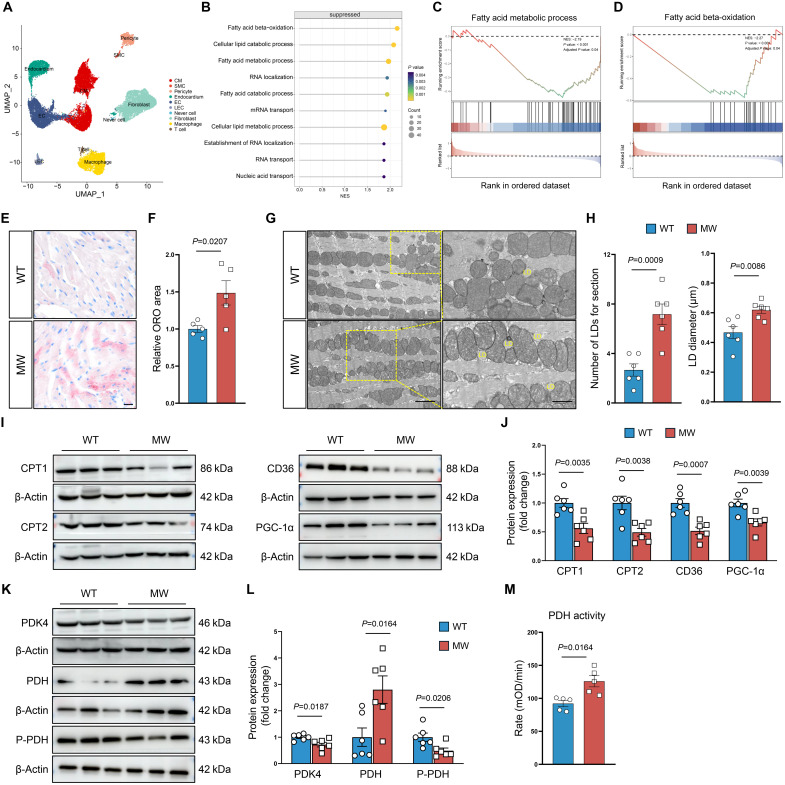
Piezo1 GOF drives cardiac dysfunction by promoting myocardial lipid accumulation. (**A**) Uniform manifold approximation and projection (UMAP) visualization of snRNA-seq profiles from mouse hearts. Cardiomyocyte (CM), smooth muscle cell (SMC), endothelial cell (EC),Lymphatic endothelial cells (LEC). (**B**) Dotplot showing suppressed pathways in mut3m samples compared with wt3m samples. *x* axis represents the normalized enrichment score (NES), dot size reflects the gene count within each pathway, and color indicates the *P* value. (**C** and **D**) GSEA of the “fatty acid metabolic process” (C) and “fatty acid beta-oxidation” (D) pathway conducted in cardiomyocytes. (**E** and **F**) Representative images (E) and quantification (F) of ORO staining of cardiac tissues from male WT and MW mice at 3 months (*n* = 5). Scale bar, 20 μm. (**G**) Representative TEM images of the myocardium of male WT and MW mice at 3 months. Scale bars, 2 and 1 μm. (**H**) Quantification of the lipid droplet (LD) number and diameter in male WT and MW mice at 3 months (*n* = 6). (**I** and **J**) Representative Western blot images (I) and quantitative analyses (J) of carnitine palmitoyltransferase 1 (Cpt1), Cpt2, CD36, and peroxisome proliferator-activated receptor-gamma coactivator 1 alpha (Pgc-1α) in cardiac tissues from male WT and MW mice at 3 months (*n* = 6). (**K** and **L**) Representative Western blot images (K) and quantitative analyses (L) of pyruvate dehydrogenase kinase 4 (Pdk4), Pdh, and P-Pdh in cardiac tissues from male WT and MW mice at 3 months (*n* = 6). (**M**) Pdh activity in cardiac tissues from male WT and MW mice at 3 months (*n* = 5). *P* values were determined by GSEA using permutation tests and adjusted using the Benjamini-Hochberg correction method [(B) to (D)]. Two-tailed unpaired Student’s *t* test was used [(F), (H), (J), (L), and (M)]. The number of samples in each group is indicated by *n*. The data are presented as the means ± SEM.

We next performed small nuclear RNA sequencing (snRNA-seq) of the hearts of 3-month-old mice (fig. S8A), which had no iron overload, to investigate the mechanism by which Piezo1 GOF affects cardiac function. Single-cell RNA sequencing (scRNA-seq) revealed the presence of 10 major cell clusters according to the expression of marker genes identified from the literature and the CellMarker database (Fig. 5A and fig. S8B) (*24*). As anticipated, cardiomyocytes from mutant mice presented increased expression of the heart failure markers Nppa and Nppb (fig. S8C). Gene set enrichment analysis (GSEA) enrichment analysis of cardiomyocytes specifically revealed that genes up-regulated by the Piezo1 GOF mutation were predominantly associated with cell-cell junctions and membrane depolarization (fig. S8D), whereas genes down-regulated by the mutation were mainly linked to lipid metabolism pathways (Fig. 5B). Specifically, the fatty acid metabolic process and fatty acid β-oxidation were significantly suppressed, with mitochondria identified as the primary site of fatty acid β-oxidation (*25*), indicating mitochondrial dysfunction (Fig. 5, C and D). Consistent with the bioinformatic results, Oil Red O (ORO) staining, used to visualize lipids and triglycerides, demonstrated increased intramyocardial lipid accumulation in the hearts of Piezo1^D674Y^ mice (Fig. 5, E and F). This finding was further corroborated by transmission electron microscopy (TEM), which revealed increases in both the number and diameters of lipid droplets in cardiomyocytes from Piezo1^D674Y^ mice (Fig. 5, G and H). The results of TEM also show the impaired mitochondrial morphology and disarrayed sarcomere. In addition, C11 Boron dipyrromethenes (BODIPY) 581/591 and BODIPY 493/503 staining revealed increased lipid peroxidation and lipid accumulation in cardiomyocytes isolated from mice with Piezo1 GOF mutation (fig. S9, A to D), possibly resulting from the reduced utilization of exogenous fatty acids to drive maximum respiration (fig. S9, E to G). Lipid metabolism relies on a delicate balance between fatty acid synthesis, uptake, and oxidation. Genes involved in lipid metabolism were significantly down-regulated in the hearts of mice with Piezo1 GOF mutation at the protein and mRNA levels (Fig. 5, I to L, and fig. S8, E and F). Furthermore, compared with those of WT mice, the hearts of mice with Piezo1 GOF mutation presented greater pyruvate dehydrogenase (Pdh) activity (Fig. 5M). This phenotype was successfully rescued by GsMTx (fig. S10, A to D). Conversely, Piezo1 overactivation by Yoda1 accelerated abnormal lipid deposition (fig. S10, E to H). These data indicate that Piezo1 GOF impairs lipid metabolism, thereby driving cardiomyopathy.

### Foxo3 prevents myocardial lipid accumulation and cardiomyopathy development in the presence of Piezo1 GOF mutation

**Fig. 6.**
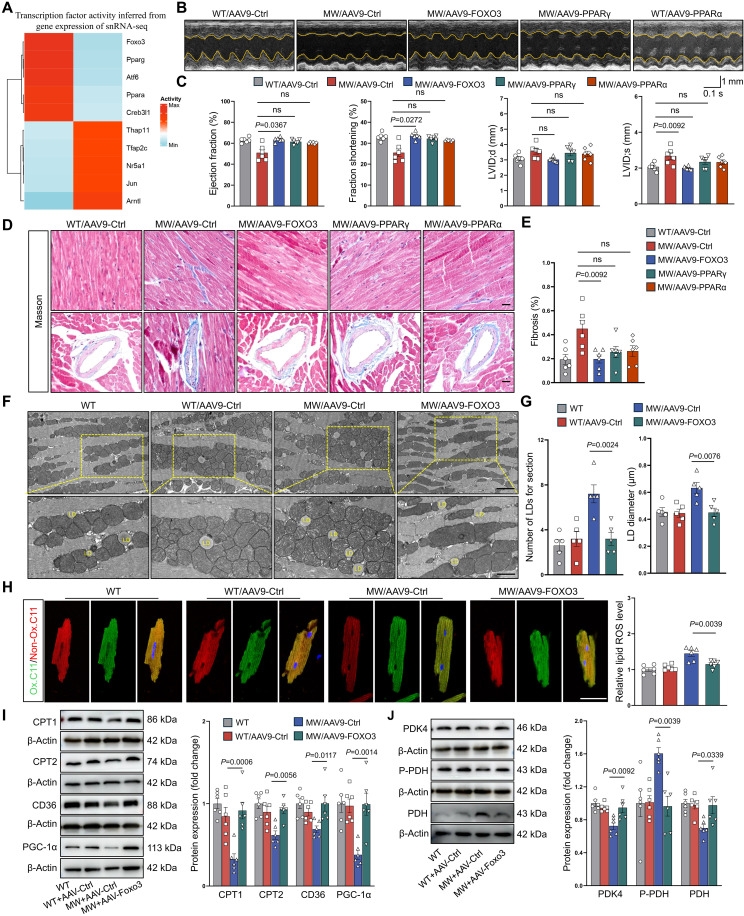
Foxo3 ameliorates cardiolipotoxicity and cardiac dysfunction in mice with Piezo1 GOF mutation. (**A**) The activity of the top five TFs in male WT and MW mice at 3 months. (**B**) Representative M-mode echocardiographic images of male WT and MW mice injected with indicated AAV9 vector. (**C**) Echocardiographic analysis of the EF, FS, LVID;d, and LVID;s of male WT and MW mice injected with indicated AAV9 vector (*n* = 6). (**D** and **E**) Representative images (D) and quantification (E) of Masson’s trichrome staining of cardiac tissues from male WT and MW mice injected with indicated AAV9 vector (*n* = 6). Scale bars, 20 μm. (**F**) Representative images of myocardial TEM images from male WT and MW mice injected with indicated AAV9 vector. Scale bars, 2 and 1 μm. (**G**) Quantification of the lipid droplet number and diameter in male WT and MW mice injected with indicated AAV9 vector (*n* = 5). (**H**) Representative images and quantitative analysis of C11-BODIPY–stained cardiomyocytes from male WT and MW mice injected with indicated AAV9 vector (*n* = 6). Scale bar, 20 μm. (**I**) Representative Western blot images and quantitative analyses of cardiac Cpt1, Cpt2, CD36, and Pgc-1α protein expression in male WT and MW mice injected with indicated AAV9 vector (*n* = 6). (**J**) Representative Western blot images and quantitative analyses of cardiac Pdk4, Pdh, and P-Pdh protein expression in male WT and MW mice injected with indicated AAV9 vector (*n* = 6). One-way ANOVA with Tukey’s test [(C), (E), and (G) to (J)], or Welch test (C) were used for the comparison of multiple groups. The number of samples in each group is indicated by *n*. The data are presented as the means ± SEM. Ctrl, control.

Deep analysis of snRNA-seq identified transcription factors (TFs) with the highest activity levels in cardiomyocytes from both Piezo1^D674Y^ and WT mice (Fig. 6A and fig. S8G). Among the top TFs identified, Foxo3, peroxisome proliferator–activated receptor α (Pparα), and Pparγ are known regulators of lipid metabolism and thus were prioritized for functional investigation. The mice were injected with adeno-associated virus 9 (AAV9) vectors encoding Foxo3, Pparα, and Pparγ at 8 weeks of ages. Echocardiography was performed at 12 weeks of age, after which hearts were collected for histological and molecular analyses. The successful overexpression was confirmed by Western blot (fig. S11). Echocardiography revealed that only Foxo3 effectively reversed the impairment of cardiac function in mice with Piezo1 GOF mutation (Fig. 6, B and C, and table S9). Furthermore, Foxo3 effectively prevented cardiac fibrosis (Fig. 6, D and E), alleviated lipid metabolic dysfunction (Fig. 6, F to H), and normalized the expression of genes involved in the lipid metabolism pathway (Fig. 6, I and J). In summary, Foxo3 acts as a downstream TF of Piezo1.

We then explored how Piezo1 GOF inactivates Foxo3. *Piezo1* overactivation was found to activate CaMKII in endothelial cells (*26*), and CaMKII is known to regulate Foxo3 expression during aging (*27*, *28*). We therefore assumed the existence of a Piezo1-CaMKII-Foxo3 signaling pathway in our mice with Piezo1 GOF mutation. Consistently, we observed elevated levels of activated phosphorylated CaMKII, accompanied by a reduction in total Foxo3 protein levels and an increase in the level of the inactive phosphorylated form of Foxo3 (fig. S12A). Immunofluorescence staining of heart sections and isolated cardiomyocytes revealed that Piezo1 GOF decreased the nuclear localization of Foxo3 (fig. S12, B and C). Besides, addition of Yoda1 to cardiomyocytes from Piezo1 GOF decreased Foxo3 nuclear localization (fig. S12D), and inhibition of CaMKII enhanced Foxo3 nuclear localization (fig. S12E). The results above further confirmed the negative regulatory effect of Piezo1 on FOXO3 signaling. In conclusion, the CaMKII–Foxo3 axis mediates the effects of PIEZO1 GOF on cardiomyocyte lipid metabolism.

### Cardiomyocyte-specific *Piezo1* overexpression induces lipotoxicity, promoting the development of cardiomyopathy

**Fig. 7.**
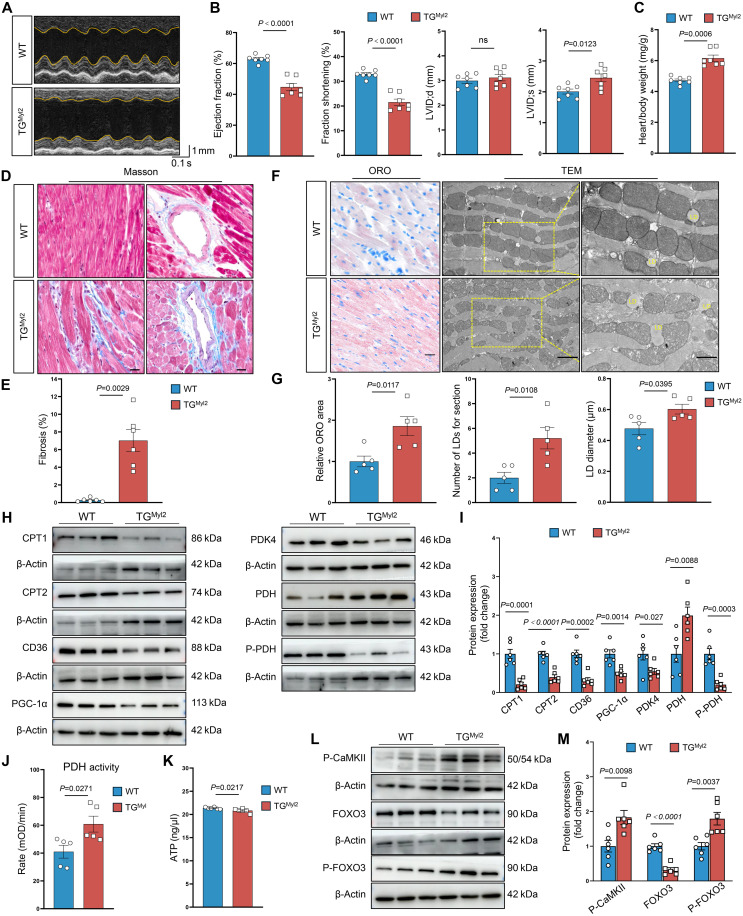
Cardiomyocyte-specific *Piezo1* overexpression impairs cardiac function and cardiac lipid metabolism. (**A**) Representative M-mode echocardiographic images of male WT and *Piezo1*-TG^*Myl2*^ (TG^*Myl2*^) mice at 4 weeks. (**B**) Echocardiographic analysis of the EF, FS, LVID;d, and LVID;s in male WT and TG^*Myl2*^ mice at 6 weeks (*n* = 10). (**C**) HW/BW ratios of male WT and TG^*Myl2*^ mice at 6 weeks (*n* = 6). (**D** and **E**) Representative images (D) and quantification (E) of Masson’s trichrome staining of cardiac tissues from male WT and TG^*Myl2*^ mice at 6 weeks (*n* = 6). Scale bar, 20 μm. (**F**) Representative images of ORO staining and TEM of cardiac tissues from male WT and TG^*Myl2*^ mice at 6 weeks. Scale bars, 20 μm. (**G**) Quantification of ORO staining, lipid droplet number, and diameter in male WT and TG^*Myl2*^ mice at 6 weeks (*n* = 5). (**H** and **I**) Representative Western blot images (H) and quantitative (I) of Cpt1, Cpt2, CD36, Pgc-1α, Pdk4, Pdh, and P-Pdh in cardiac tissues from male WT and TG^Myl2^ mice (*n* = 6). (**J**) Pdh activity in cardiac tissues from male WT and TG^Myl2^ mice at 6 weeks (*n* = 5). (**K**) Concentrations of adenosine 5′-triphosphate (ATP) in cardiac tissues from male WT and TG^*Myl2*^ mice at 6 weeks (*n* = 6). (**L** and **M**) Representative Western blot images (L) and quantitative (M) of P-CaMKII, Foxo3, and P-Foxo3 in cardiac tissues from male WT and TG^*Myl2*^ mice (*n* = 6). Two-tailed unpaired Student’s *t* test was used [(B), (C), (E), (G), (I), (J), (K), and (M)]. The number of samples in each group is indicated by *n*. The data are presented as the means ± SEM.

We examined the phenotypes of two mice with cardiomyocyte-specific *Piezo1* overexpression to extend our findings to other GOF mutations (figs. S13, A to E, and S16, A to E). Transgenic mice expressing *Piezo1* under the control of *Myl2* promoter (hereafter referred to as TG^Myl2^ mice) were significantly smaller in size and exhibited an enlarged heart (figs. S13, G and H, and S14, B and E) and a higher HW/BW ratio at 6 weeks (Fig. 7C and fig. S14, C and D). all TG^*Myl2*^ mice survived to adulthood (figs. S13F and S14A). At 6 weeks of age, the TG^Myl2^ mice presented impaired cardiac function, enlarged ventricular chambers (Fig. 7, A and B; figs. S13, I and J, and S14, F to I; and tables S10 and S11), and severe myocardial fibrosis (Fig. 7, D and E, and fig. S14J). The TG^*Myl2*^ mice presented abnormal lipid metabolite accumulation (Fig. 7, F to K, and fig. S14K) and lipid metabolism dysfunction. Specially, P-CaMKII and P-Foxo3 were expressed at higher levels in the hearts of *Piezo1* TG^*Myl2*^ mice than in those of Piezo1-TG^loxp-stop-lopx^ mice (Fig. 7, L and M), accompanied by reduction of Foxo3 nuclear localization (fig. S15A). TG^*Myl2*^ mice presented the same phenotype as mice with Piezo1 GOF mutation.

In parallel, we studied mice with *Myh6*-Cre–induced ventricle-specific *Piezo1* overexpression (designated as TG^*Myh6*^ mice). Notably, unlike TG^*Myl2*^ mice, all TG^*Myh6*^ mice died before reaching 8 weeks of age, whereas all *Piezo1*-TG^loxp-stop-lopx^ mice (WT mice in the figures) survived to adulthood (figs. S16F and S18A). At 4 weeks of age, the TG^*Myh6*^ mice presented impaired heart function and lipid metabolism defects, which were analogous to those of the TG^*Myl2*^ mice (figs. S16, S17, and S18; and tables S12 and S13). Iron overload was not observed in the hearts or livers of TG^*Myl2*^ and TG^*Myh6*^ mice (fig. S19). These data indicate that *Piezo1* overexpression dysregulates lipid metabolism, leading to cardiomyopathy besides iron overload, in a manner consistent with that observed in mice with Piezo1 GOF mutation.

## DISCUSSION

The unexplained cardiomyopathy observed in PIEZO1 GOF mutation carriers prompted us to investigate the etiology of PIEZO1-associated cardiomyopathy. By integrating evidence from humans and animal studies, we confirmed an important role for PIEZO1 GOF in the pathogenesis of cardiomyopathy. Mechanistic investigations revealed that Piezo1 GOF mutation increases calcium influx, contributing to cardiolipotoxicity and the development of cardiomyopathy through calcium signal–induced activation of the CaMKII-Foxo3 axis. This study provides critical insights for molecular diagnosis, prognosis prediction, and the development of potential therapeutic strategies for managing this condition.

### Evidence linking PIEZO1 GOF mutations to cardiomyopathy

Recent advances in understanding the role of PIEZO1 in iron overload and excitation-contraction coupling have raised critical questions regarding the etiology of PIEZO1-related cardiomyopathy (*7*). In the present study, the follow-up of a patient with a rare form of cardiomyopathy complicated by iron overload revealed a poor association between cardiac function and myocardial iron deposition. In mice harboring the Piezo1 GOF mutation and treated with a Piezo1 activator and in mice with cardiac-specific *Piezo1* overexpression, cardiac dysfunction was induced before obvious cardiac iron overload. These findings suggest the role of *Piezo1* GOF in cardiac defects beyond its established role in iron overload.

The previously reported PIEZO1 M2241R GOF variant, which causes mild cardiac hypertrophy and fibrosis in homozygous mice (*29*), provides an important comparison point. In contrast, our D669Y variant appears lethal in homozygosity, as no homozygous mice were obtained. This phenotypic difference likely reflects a greater functional impact of D669Y mutation. Supporting this, M2241R is more frequently observed in the general population (allele frequency of ~0.0035% in UK Biobank), whereas D669Y was not detected, suggesting higher pathogenicity. Such variation is consistent with known features of genetic cardiomyopathies, where different mutations in the same gene can result in distinct disease types depending on their functional severity (*30*, *31*).

Notably, approximately one-third of individuals of African descent are estimated to carry a mild *PIEZO1* GOF allele (*32*). In parallel, individuals of African ancestry have been reported to exhibit a 2.6-fold higher risk of developing idiopathic DCM compared to individuals of European descent, and DCM remains a major cause of heart failure throughout Africa, where cardiomyopathy is otherwise relatively uncommon (*33*). For decades, it has been hypothesized that region-specific genetic factors may contribute to this disparity in disease burden (*33*, *34*). In this study, we provide evidence supporting a pathogenic role for PIEZO1 GOF in DCM. Given the high prevalence of *PIEZO1* GOF alleles in African populations, it is plausible that these variants may contribute to the elevated incidence of DCM in this group—a hypothesis that merits further investigation.

### Impaired lipid metabolism as the mechanism underlying *PIEZO1* GOF–induced cardiomyopathy

PIEZO1 is a Ca^2+^-permeable mechanosensitive ion channel (*7*). Here, we observed conformational changes that increased intracellular Ca^2+^ influx upon PIEZO1 GOF mutation and revealed that PIEZO1 GOF mutations promote lipid accumulation and cardiomyopathy. While the role of PIEZO1 in lipid metabolism has been studied in other tissues, such as the vasculature and duodenum (*35*), its role in the heart has not been reported previously. In the human heart, which relies heavily on FAs for energy to sustain basal metabolism and contraction (*36*, *37*), cardiac lipotoxicity and dysfunction can develop upon the accumulation of excess lipids (*38*). Our findings highlight that PIEZO1 GOF mutations alter cardiac lipid metabolism, providing the first evidence of the role of PIEZO1 in cardiomyopathy through lipid metabolic dysregulation.

We further identified that the enhanced Ca^2+^ influx upon GOF Piezo1 activates calcium-dependent kinases (e.g., CaMKII) and phosphorylates Foxo3. Foxo3 is known to regulate cell metabolism, mitochondrial function, and energy homeostasis and has been shown to influence lipid metabolism in peripheral tissues and the central nervous system (*39*–*41*), suggesting its potential involvement in lipid metabolism dysregulation in the heart. Our data revealed that Foxo3 overexpression significantly reduced lipid accumulation and alleviated cardiomyopathy in the presence of Piezo1 GOF. CaMKII, activated by Ca^2+^, phosphorylates Foxo3 to regulate its expression and transcriptional activity in a context-dependent manner (*28*, *42*). Given that elevated Ca^2+^ levels can induce mitochondrial overload, leading to a shift in cardiac energy utilization from fatty acids to glucose and disrupting normal lipid metabolism, this raises another potential mechanism underlying Piezo1 GOF and cardiac lipid dysfunction, warranting further investigation.

### Implications for clinical care

In this study, we propose that the pathogenesis of PIEZO1-related cardiomyopathy is multifactorial. Notably, the use of the iron chelator deferasirox, in conjunction with anti–heart failure therapy, reduces serum ferritin levels—a surrogate marker for iron overload—and simultaneously improves cardiac function. Although the cardiac function is not entirely correlated with myocardial iron deposition, it is possible that changes in systemic iron levels contribute to secondary benefits in cardiac outcomes. In addition to myocardial iron deposition, PIEZO1 GOF variants may impair red blood cell turnover and contribute to anemia, which is often compensated and subclinical, yet may still contribute to the development of cardiomyopathy over time. Therefore, therapies that combine iron chelation with β-blockers, angiotensin converting enzyme (ACE) inhibitors, ARNI, and sodium-glucose cotransporter-2 (SGLT2) inhibitors—not iron chelation alone—appear essential for achieving meaningful cardiac recovery in affected individuals.

It is important to emphasize that the cardiac impairment in GOF PIEZO1 carriers with iron overload and acute decompensated heart failure is reversible, changing traditional understanding of prognoses for iron overload cardiomyopathy. Traditionally, patients with transfusion-related iron overload cardiomyopathy who present with reduced LVEF or decompensated heart failure have a median survival of less than 1 year (*43*). In contrast, our clinical case involving a PIEZO1^D669Y^ carrier demonstrated complete normalization of cardiac function despite severe myocardial iron deposition and reduced LVEF at presentation. This finding has important prognostic implications, particularly as a similar HX case with DCM and iron overload was recently reported, in which the patient was considered for heart transplantation due to presumed end-stage heart failure (*13*). These observations suggest that the natural history of iron overload cardiomyopathy may differ in the presence of PIEZO1 GOF mutations and highlight the need for genotype-informed approaches in treatment and drug development.

Our findings have substantial implications for the development of therapeutic strategies targeting PIEZO1 mutations, which are systemic genetic alterations with effects across multiple cell types. PIEZO1 GOF mutations induce aberrant increases in intracellular Ca^2+^ concentrations in erythrocytes, leading to activation of the Gardos channel, potassium efflux, cellular dehydration, and consequent erythrocyte pathology (*32*). In macrophages, PIEZO1 GOF dysregulates hepcidin expression, contributing to systemic iron overload (*16*). In cardiomyocytes, increased PIEZO1-mediated Ca^2+^ signaling impairs lipid metabolism, ultimately contributing to heart failure. Therefore, systemic pharmacological interventions that target PIEZO1 activity across affected tissues are essential. Benzbromarone, a clinically used agent, also functions as a PIEZO1 inhibitor (*44*), making its potential cardiac effects worthy of further investigation.

### Limitations

HX or iron overload resulting from PIEZO1 GOF mutations is relatively rare. In larger populations, investigating the relationship between PIEZO1 mutations and heart failure is particularly challenging. In this study, we confirmed that PIEZO1 contributes to cardiomyopathy through an iron-independent mechanism. However, myocardial iron accumulation markedly affects cardiac function, particularly in late-stage disease—a concept that was not specifically addressed in this study.

## MATERIALS AND METHODS

### Patient information and ethics

The whole-exome sequencing and Sanger sequencing were performed by SINO-US Diagnostics. Patient echocardiography data, peripheral blood test results, and cardiac magnetic resonance images were collected at Qilu Hospital. The proband was treated with medication under physician supervision for a duration of ~38 months. As an observational study, this research adhered to standard diagnostic and treatment protocols without intervention. Written informed consent was provided by the proband. All protocols were performed in compliance with Qilu Hospital, Shandong University (KYLL-202409-045).

### Animals

The animal procedures and the use of animals were approved by the Ethics Committee and Scientific Investigation Board of Qilu Hospital, Shandong University, China (DWLL-2022-103). These practices comply with the guidelines outlined in the Guide for the Care and Use of Laboratory Animals, published by the National Institutes of Health (NIH) in the United States. The mice were housed at a constant temperature (20° to 22°C) and humidity (5060%) in a specific-pathogen-free animal facility on a 12-hour light/dark cycle.

Human mutations were mapped onto the mouse genome to identify mouse mutations for the experiment. The human p.669D variant was mapped to the murine p.674Y variant. To generate Piezo1 mutant mice, we targeted exon 16: p.D674Y of the piezo1 gene via CRISPR-Cas9 technology according to the standard protocol. Heterozygous piezo1 mutant mice were used for the experiments unless mentioned otherwise.

*Piezo1*-TG-flox-stop-flox mice (Shanghai Model Organisms Center, Inc., catalog no. CM-KI-232382) were mated with *Myh6*-Cre and *Myl2*-Cre-IRES-EGFP mice to generate cardiac-specific *Piezo1* transgenic mice [*Piezo1*-TG-flox-stop-flox/*Myh6*-Cre (*Piezo1*-TG^*Myh6*^) and *Piezo1*-TG-flox-stop-flox/*Myl2*-Cre-IRES-EGFP (*Piezo1*-TG^*Myl2*^) mice].

Male mice were used for all experiments unless otherwise stated. Female mice were included only in cardiac function analyses. Littermates were randomly selected by genotype.

### Mouse echocardiography

Mouse echocardiographic parameters were measured via a high-resolution ultrasound imaging system (Vevo3100, Visual Sonics) by an investigator blinded to the genotypes and treatment groups. The parasternal long-axis view was used to obtain M-mode images for analysis of the LVEF, FS, and other cardiac functional parameters. The apical four-chamber view was used for tissue Doppler imaging and pulse-wave Doppler imaging for analysis of the myocardial velocity and blood flow velocity, respectively. During the entire process, the mice were anesthetized with 1.2 to 1.5 vol % of isoflurane, and the heart rate was maintained at more than 450 beats per minute.

### Measurement of mouse body weight, heart weight, and tibia length

The mice were weighed and then sacrificed. The hearts were rapidly removed, trimmed to remove the major blood vessels, and then weighed. After the mice were euthanized, the tibias were dissected, and the tibia lengths were measured.

### Drug administration to animals

In a study of drug administration, male mice received daily intraperitoneal injections of the drug from 8 weeks of age for 30 days. At 12 weeks of age, echocardiography was performed on the mice immediately before they were euthanized. Hearts were then harvested for further analysis. Yoda1 (HY-18723, MCE) was dissolved in anhydrous dimethyl sulfoxide (DMSO; D8371, Sigma-Aldrich) to a concentration of 10 mg/ml according to the manufacturer’s manual. For the animal experiments, the stock solution was further diluted in corn oil at a 1:9 ratio, and the mice were treated with Yoda1 (3 mg/kg) or an equivalent amount of DMSO dissolved in corn oil by intraperitoneal injection every day for 30 days. For the GsMTx4 rescue experiment, GsMTx4 (HY-P1410, MCE) was dissolved in Milli-Q water and administered (1 mg/kg) by intraperitoneal injection into mutant mice for 30 days.

### AAV9 injection

An AAV9 vector (OBiO Technology) carrying the Foxo3, Pparγ, and Pparα coding sequences under the control of the cardiomyocyte-specific promoter troponin T2 (*cTnT*) (AAV9-*cTnT-Foxo3*, AAV9-*cTnT-Pparγ*, and AAV9-*cTnT-Pparα*) or a control virus (AAV9-*cTnT*-GFP) was injected into the tail vein of 8-week-old mice at a dose of 5 × 10^11^ vector genomes. The hearts were harvested at 12 weeks of age for subsequent analysis.

### Iron concentration measurement

Total iron levels were measured using an Iron Assay Kit (MAK025, Sigma-Aldrich) according to the manufacturer’s manual. In brief, tissue was collected from mice and homogenized in the iron assay buffer, and the resulting supernatant was centrifuged. Serum or tissue supernatant was added directly to a transparent 96-well plate for chemical reactions to measure ferrous (Fe^2+^) iron, total iron, or ferric (Fe^3+^) iron (total iron ferrous iron). In this assay experiment, iron was released by the addition of an acidic buffer, and the released iron reacted with a chromagen, resulting in the formation of a colorimetric (593 nm) product, with the levels of the product being proportional to the amount of iron present. Standard curves were prepared using iron solutions provided by the kit. All colorimetric readings were obtained with a Cytation 5 multimode plate reader (BioTek, Winooski).

### Transferrin saturation

Transferrin saturation was detected by a Total Iron-Binding Capacity (TIBC) and Serum Iron Assay Kit (AB239715, Abcam) following the manufacturer’s instructions. Briefly, samples, standards, and controls were prepared and added to the wells, followed by the addition of reagents as specified in the kit. The absorbance values were measured at 570 nm and analyzed on a Cytation 5 multimode plate reader (BioTek, Winooski). A standard curve was generated using the iron solutions provided in the kit. The percentage of transferrin saturation was determined by calculating the serum iron level relative to the TIBC.

### Measurement of ferritin levels in tissues

The ferritin concentration in mouse serum was measured with a Mouse Ferritin ELISA Kit (E-EL-M0491, Elabscience). Briefly, samples were added to an ELISA plate well and incubated for 90 min with gentle shaking. Then, the samples were immediately incubated with Biotinylated Detection Ab Working Solution for 1 hour. Horseradish peroxidase (HRP) Conjugate Working Solution and Substrate Reagent were incubated with the samples after extensive washing. Last, stop solution was added to terminate the reaction. The absorbance at 450 nm was determined with a Cytation 5 multimode plate reader (BioTek, Winooski). A standard curve was prepared from the 4PL exponential sigmoidal.

### Measurement of ATP levels, PDH activity and Piezo1 concentrations

Cardiac adenosine 5′-triphosphate (ATP) levels were measured using a commercial kit (MAK-190, Sigma-Aldrich) according to the manufacturer’s instructions. PDH activity was measured in protein samples extracted from cardiac tissues using a kit (AB109902, Abcam). The concentration of Piezo1 in cardiac tissues was determined via an ELISA kit (EK4579, Signalway Antibody).

### Adult mouse cardiomyocyte isolation

Adult cardiomyocytes were isolated from Piezo1 mutant, Piezo1-TG, and littermate control mice. In brief, the mice were sacrificed by cervical dislocation, and the chest was opened. The heart was exposed and rinsed in cold perfusion buffer (PB) [pH 7.4; in mM: NaCl 137, glucose 15, Hepes 20, KCl 4.9, MgCl_2_ 1.2, NaH_2_PO_4_ 1.2, taurine 5, and 2,3-butanedione monoxime (BDM) 10] and then immediately mounted on a Langendorff perfusion system. The hearts were then perfused with 37°C oxygenated Ca^2+^-free PB until the blood was completely clear, followed by incubation with collagenase buffer [PB containing type II collagenase (0.7 mg/ml; LS004176, Worthington), type XIV protease (0.1 mg/ml; P5147, Sigma-Aldrich), bovine serum albumin (BSA) (1 mg/ml; A3912, Sigma-Aldrich) and 50 μM Ca^2+^] for 15 to 30 min. Once the hearts became soft, the heart chambers were separated, gently pulled into 1-mm pieces via a forceps and agitated via blunt-tipped transfer pipettes in stop buffer [PB containing BSA (1 mg/ml) and 50 μM Ca^2+^]. The cells were passed through a 100-μm cell strainer and subjected to Ca^2+^ gradient recovery [PB containing BSA (1 mg/ml) and 0.25, 0.35, 0.52, or 1 mM Ca^2+^]. The precipitate, which contained cardiomyocytes, was incubated with 10 μM autocamtide-2-related inhibitory peptide (HY-P0214A, MCE) for 1 hour or 5 μM Yoda1 for 30 min and subsequently cultured in minimum essential medium supplemented with 10 mM BDM for further experiments.

### RNA extraction and quantitative RT-PCR

Total RNA was extracted via TRIzol reagent (15596026CN, Thermo Fisher Scientific) according to the manufacturer’s instructions and reverse transcribed to cDNA via a PrimeScript RT Reagent Kit (R211, Vazyme). Quantitative polymerase chain reaction (PCR) was performed with SYBR Green Master Mix (Q711, Vazyme) on the ABI Prism 7500 system. The ΔΔCt method was used to calculate the relative changes in gene expression. The sequences of the primers used for reverse transcription (RT)–PCR are listed in table S14.

### Histological analysis

Excised hearts or livers were quickly rinsed in phosphate-buffered saline (PBS) buffer and incubated in 4% paraformaldehyde for at least 24 hours at room temperature. The samples were dehydrated with ethanol, mounted in paraffin, and sectioned at a thickness of 5 μm. The sections were subjected to hematoxylin and eosin staining (G1120, Solarbio), Masson’s trichrome staining (G1346, Solarbio), wheat germ agglutinin staining (L4895, Sigma-Aldrich), and Perls Prussian blue staining (G1428, Solarbio) according to the recommended procedures to visualize the tissue architecture. All images were taken with an Olympus VS200 microscope and viewed with OlyVIA software.

### Immunocytochemistry

Sections and fixed cells were blocked with 5% BSA, followed by incubation overnight at 4°C with primary antibodies diluted in 1% BSA. Then, the samples were incubated with Alexa Fluor–conjugated secondary antibodies for 1 hour at room temperature, and the nuclei were stained with 4′,6-diamidino-2-phenylindole (DAPI; AB104139, Abcam). Immunofluorescence images were visualized via a confocal microscope (SP8, Leica) and LAS X software.

### Western blotting

Total protein was extracted from tissues by lysing them in radioimmunoprecipitation assay buffer containing a protease/phosphatase inhibitor cocktail. For immunoblot analysis, protein samples were resolved by SDS–polyacrylamide gel electrophoresis and transferred to polyvinylidene difluoride membranes (IPVH00005, Millipore). The membranes were blocked in 5% milk solution for 1 hour and incubated with primary antibodies overnight at 4°C. The membranes were subsequently incubated with HRP-conjugated secondary antibodies. The protein bands were detected using enhanced chemiluminescence detection reagent (WBKLS0500, Millipore). The antibodies used for Western blotting are listed in table S15.

### Ca^2+^ spark measurements

Freshly isolated cardiomyocytes were incubated with 5 μM Fluo-4 AM (F14201, Invitrogen) for 10 min at 37°C. The cells were plated on laminin-coated 35-mm glass-bottom confocal dishes and scanned via a 488-nm laser in confocal line-scan mode. Automated analysis of the line-scan images of Ca^2+^ sparks was performed via ImageJ software (*45*).

### Ca^2+^ transient measurement

The freshly isolated cardiomyocytes were used to measure intracellular Ca^2+^ handling using the IonOptix system (IonOptix Corporation, US). In brief, cardiomyocytes were incubated with 2 μM Fura-2 AM (F1201, Invitrogen) for 20 min at room temperature and then perfused with Ca^2+^ Tyrode’s solution and stimulated at a frequency of 1 Hz by MyoPacer EP Cell Stimulator (IonOptix). Ca^2+^ transients were recorded using a Ca^2+^-recording system. Data analysis was performed with homemade routines using IonWizard software version 7.4 (IonOptix).

### Computational analysis of the Piezo1^D674Y^ mutant protein

#### Protein preparation

The mouse Piezo1 trimer structure was retrieved from the Protein Data Bank under the RCSB PDB ID 6B3R 1. While there were numerous missing residues in the nonmembrane region in the cryo–electron microscopy structure of Piezo1, it is anticipated that these gaps have a minimal influence on the overall structure owing to their flexibility and distance from the ion channel. In addition, predictions by AlphaFold2 indicated disorder in these missing residues, further justifying not repairing them. To address the charge balance resulting from the absence of these residues in the extramembrane region, *N*-methyl and acetyl capping groups (NME and ACE residues) were used to cap the carbon and nitrogen termini, respectively.

### Protein-membrane complex construction

The protein-membrane complex was constructed using the CHARMM-GUI web server at www.charmm-gui.org. The Piezo1 trimer was selected from the OPM (Orientations of Proteins in Membranes) database to define the protein-membrane contact region, while the bilayer membrane was exclusively composed of dipalmitoylphosphatidylcholine (DPPC) phospholipid molecules. As the Piezo1 trimer spans a curved membrane, constructing it through CHARMM-GUI was not straightforward. Initially, a flat membrane was used to construct a single monomer of Piezo1, which later served as a template for assembling the Piezo1 trimer by aligning each Piezo1 monomer to the individual chains in the trimer. Conflicting phospholipids were removed, followed by the elimination of phospholipid pairs, creating conflicts. Subsequently, additional phospholipids were added to extend the system at the same horizontal level as the outermost phospholipids in contact with the Piezo1 trimer.

### MD simulation

Given that the “curved” membrane comprised three flat membranes, preoptimization was essential. The initial phase of the simulation involved minimizing the bilayer membrane, particularly focusing on the curved region. Upon conducting over one hundred thousand minimization steps, the construction of the curved membrane was completed. The system subsequently underwent a heating process using the Langevin thermostat in the NVT ensemble, increasing the temperature from 0 to 300 K over 200 ps. This was followed by an equilibrium phase at 300 K and 1 atm pressure. Pressure control was achieved with a Monte Carlo anisotropic barostat, with independent control in the *x* and *y* directions by an isotropic barostat, whereas the *z* direction remained unaffected. Using the GPU-accelerated pmemd.cuda module integrated in Amber205, 450-ns long-term MD simulations with an anisotropic barostat were performed for both the WT Piezo1 trimer and the Piezo1^D674Y^ trimer.

### Calcium ion translocation analysis

Steered MD simulations were used to investigate the translocation velocity of Ca^2+^ ions across the phospholipid membrane. The simulations were performed using GROMACS 2021.5, using CHARMM36 and cgenff force field for the protein and small molecules. The whole system combining the phospholipid bilayer and small molecules (A and B) was solvated in a TIP3P water box and then neutralized with Na^+^ ions. Following energy minimization and equilibration of temperature and pressure, the production phase of the MD simulation was conducted under normal pressure and temperature (NPT) conditions, with hydrogen bond constraints and a 2-fs integration time step. The simulation concluded once the small molecules traversed the membrane by more than 30 Å, and subsequent visualization and analysis were carried out using GROMACS and VMD tools.

### Isolation of nuclei from the ventricular tissues for sequencing

Ventricular tissues were harvested from the mice and washed in precooled PBSE (PBS buffer containing 2 mM EGTA). Nuclei were isolated using GEXSCOPE Nucleus Separation Solution (Singleron Biotechnologies, Nanjing, China) according to the manufacturer’s instructions. The isolated nuclei were resuspended in PBSE to a concentration of 10^6^ nuclei per 400 μl, filtered through a 40-μm cell strainer, and counted by staining with Trypan blue. The nuclei in PBSE were stained with DAPI (1:1000) (D1306, Thermo Fisher Scientific). Nuclei were defined as DAPI-positive singlets.

### scRNA-seq library preparation

The concentrations of single-nucleus suspensions were adjusted to 3 to 4 × 10^5^ nuclei/ml in PBS. The single-nucleus suspensions were then loaded onto a microfluidic chip (GEXSCOPE Single Nucleus RNA-seq Kit, Singleron Biotechnologies), and snRNA-seq libraries were constructed according to the manufacturer’s instructions (Singleron Biotechnologies). The resulting snRNA-seq libraries were sequenced on an Illumina NovaSeq 6000 instrument with 150–base pair paired-end reads.

### Primary analysis of raw read data

The raw scRNA-seq reads were processed to generate gene expression matrixes using the CeleScope (https://github.com/singleron-RD/CeleScope) v1.9.0 pipeline. Briefly, the raw reads were first processed with CeleScope to remove low-quality reads, and Cutadapt v1.17 (*46*) was used to trim poly-A tail and adapter sequences. The cell barcode and unique molecular identifier (UMI) were extracted. Afterward, we used STAR v2.6.1a (*47*) to map the reads to the reference genome GRCm38 (ensemble version 92 annotation). UMI counts and gene counts for each cell were acquired with featureCounts v2.0.1 (*48*) software and used to generate expression matrix files for subsequent analysis. The raw sequencing data are deposited at the National Genomics Data Center (https://ngdc.cncb.ac.cn/) under project PRJCA045377.

### snRNA-seq data processing

To remove ambient RNA contamination and doublets, SoupX v1.6.2 (*49*) and DoubletFinder v2.0.4 (*50*) were used. In addition, we discarded cells (i) with fewer than 200 and more than 1500 genes, (ii) with more than 20% mitochondrial gene expression, and (iii) with more than 5% hemoglobin gene expression. The “NormalizeData” and “ScaleData” functions of Seurat v4.1.0 (*51*) software with default parameters were first used to erase the depth differences and scale the raw matrix. Principal components analysis (PCA) was then used to reduce the dimensionality of the top 2000 most variable genes, condensing them down to 50 dimensions. To remove the variation between individual samples, the Harmony v1.0 (*52*) algorithm of the RunHarmony function was used based on the PCA results. Then, the top 20 principal components were subsequently used for cell clustering via the “FindNeighbors” and “FindClusters” functions with default parameters. Uniform manifold approximation and projection (UMAP) was further used to project the cells into two dimensions for visualization. Last, the “FindAllMarkers” function was used to identify the marker genes for each cluster, which were further annotated by overlapping the cluster markers with cell-type signature genes.

### Functional enrichment analysis

To reveal the functional pathways active in cardiomyocytes from mice of different genotypes, the “FindMarkers” function was used to identify highly expressed marker genes. After the differentially expressed genes were sorted on the basis of the fold change in expression, the “gseGO” function of the R package clusterProfiler v4.9.2 (*53*) was used to perform Gene Ontology enrichment analysis.

### Analysis of TF activity

Using the R package DoRothEA v1.14.1 (*54*), we assessed the activity of TFs in cardiomyocytes from mice of different genotypes by analyzing the expression patterns of their respective target genes.

### Transmission electron microscopy

Fresh heart tissues were immediately fixed in 2.5% glutaraldehyde at 4°C overnight and subsequently fixed with 1% OsO_4_ for 1.5 hours. Then, the samples were dehydrated in graded ethanol solutions and incubated with acetone. All the samples were embedded in ethoxyline resin and cut into ultrathin sections with a Leica UC7 ultramicrotome, after which they were counterstained with uranyl acetate and lead citrate. TEM images were captured with an HT-7800 microscope (Japan Hitachi Laboratory). Mitochondria and lipid droplets were analyzed with Fiji ImageJ software (NIH, Bethesda, MD, United States).

### ORO and BODIPY lipid staining

ORO and BODIPY staining were used to assess the accumulation of lipids in cardiac tissues and cardiomyocytes. Frozen sections of cardiac tissue were subjected to ORO staining with a kit (G1262, Solarbio) according to the manufacturer’s instructions. For BODIPY lipid staining, cardiomyocytes were incubated with 10 μM BODIPY (D3922, Invitrogen) and 5 μM C11 BODIPY 581/591 (SML3717, Sigma-Aldrich) for 30 min at 37°C. Following two washes with PBS, cardiomyocytes were covered with mounting medium containing DAPI (AB104139, Abcam). The images were visualized via a Leica confocal microscope and LAS X software.

### Oxygen consumption rate measurement

The oxygen consumption rate (OCR) of cardiomyocytes was measured with a Seahorse XFe96 Analyzer (Agilent) per the manufacturer’s instructions. Freshly isolated cardiomyocytes were seeded in laminin (10 μg/ml)–coated Seahorse XF Cell Culture Microplates, energy substrates were added to the medium, and the mixture was incubated for 1 hour in a 37°C incubator without CO_2_ before the OCR was measured. For the fatty acid oxidation assay, BSA, palmitic acid, and 0.2 mM l-carnitine were added to the wells, three metabolism inhibitors were sequentially added to the plate, and the OCR/extracellular acidification rate ratio was measured. The data were analyzed via Wave software (Agilent Technologies, Inc.).

### Statistical analysis

All the data are presented as means ± SEM, and *P* < 0.05 was considered to indicate statistical significance. The normality of the data distribution was analyzed via the Shapiro-Wilk test. The homogeneity of variances was assessed using the Levene test or Brown-Forsythe test. For normally distributed data with equal variances, the groups were compared via Student’s *t* test or one-way analysis of variance (ANOVA) with Tukey’s post hoc test; for data with unequal variances, Welch’s *t* test or Welch ANOVA with Games-Howell post hoc test was used. For non-normally distributed data, the groups were compared via the Mann-Whitney test or Kruskal-Wallis test with Dunn’s post hoc test and Holm adjustment. Statistical analysis and graphing were performed with GraphPad Prism software (version 8.0). The researchers who performed data collection and analysis were blinded to the experimental conditions.

## Supplementary Materials

### The PDF file includes:

Figs. S1 to S20 Tables S1 to S15 Legends for movies S1 and S2 Legend for dataset S1 Uncropped Western blots
