# Supplementary material for: PIEZO1 gain-of-function mutation drives cardiomyopathy by disrupting myocardial lipid homeostasis besides iron overload
Source: Sci Adv. 2025 Nov 14;11(46):eady9242. doi: 10.1126/sciadv.ady9242 (PMC12617528; doi:10.1126/sciadv.ady9242)

Science Advances
AAAS

Supplementary Materials for

PIEZO1 gain-of-function mutation drives cardiomyopathy by disrupting myocardial lipid homeostasis besides iron overload

Cuiqin Fan *et al.*

Corresponding author: Feng Xu, xufengsdu@126.com; Yuguo Chen, chen919085@sdu.edu.cn; Sumei Cui, sumeicui@email.sdu.edu.cn

Sci. Adv. **11**, eady9242 (2025)
DOI: 10.1126/sciadv.ady9242

The PDF file includes:

Figs. S1 to S20
Tables S1 to S15
Legends for movies S1 and S2
Legend for dataset S1
Uncropped Western blots

Other Supplementary Material for this manuscript includes the following:

Movies S1 and S2
Dataset S1

Supplementary Materials

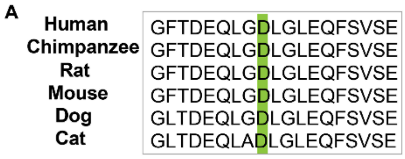

Figure S1. Species conservation analyses of *PIEZO1*^{D669Y} mutation

A, Species conservation analyses of *PIEZO1*^{D669Y} mutation

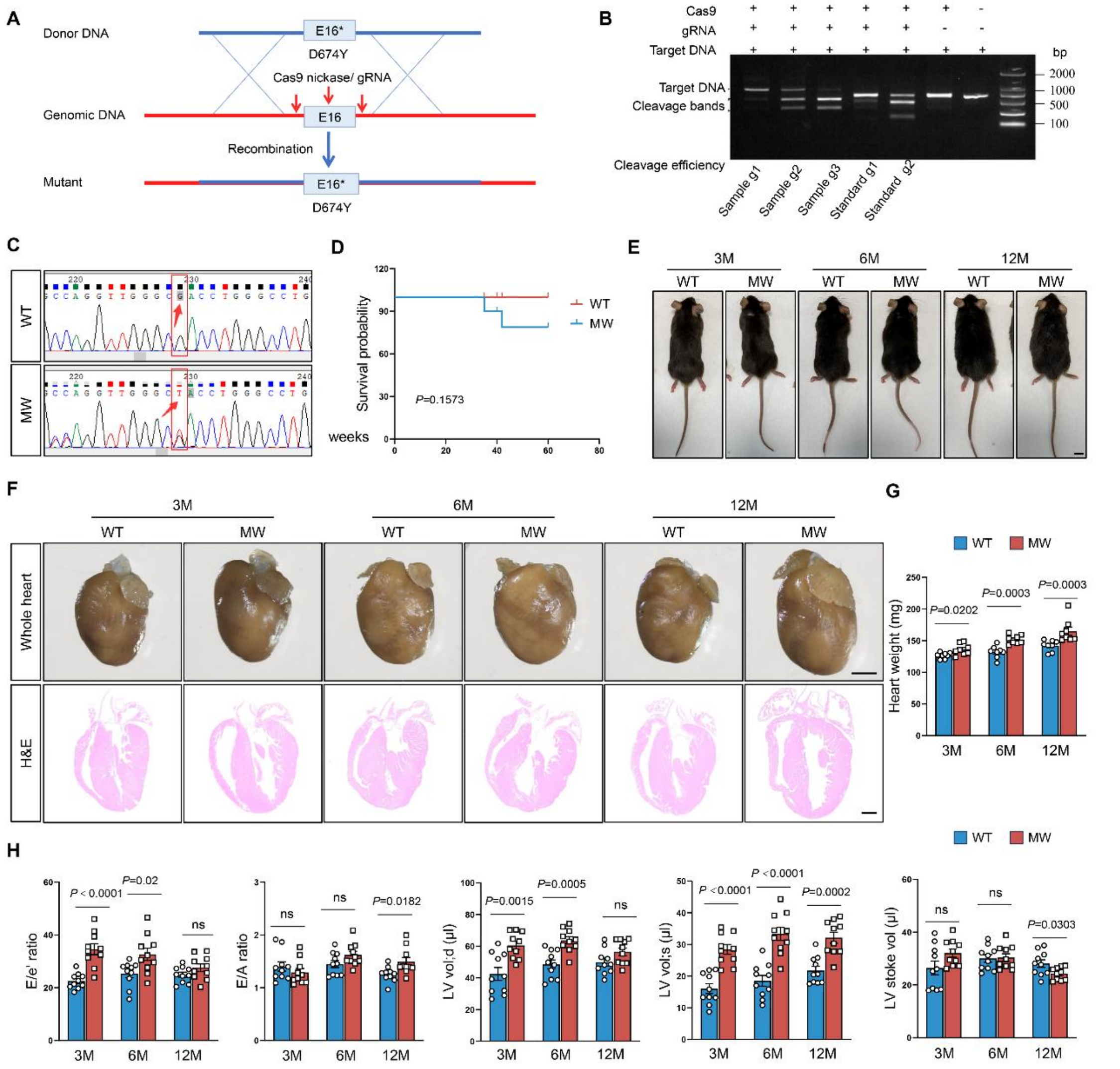

Figure S2. Generation and phenotypic analysis of heterozygous *Piezo1*^{D674Y} mice

A, Diagram of the generation of *Piezo1*^{D674Y} mice via CRISPR. **B**, Analysis of the activity of spCas91.1/gRNA by cutting DNA in vitro. **C**, Sanger sequencing results for *Piezo1*^{D674Y} mice. **D**, Survival curves of male WT and MW mice (n = 10). The Kaplan–Meier method was used to compare the data via the log-rank test. **E**, Photograph of male WT and MW mice at 3 to 12 months (scale bar = 1 cm). **F**, Representative images of whole hearts (scale bar = 2 mm) and H&E-stained longitudinal heart sections (scale bar = 1 mm) from male WT and MW mice at 3 to 12 months. **G**, Quantification of the HW of male WT and MW mice at 3 to 12 months (n = 8). **H**, Echocardiographic analysis of the flow Doppler E wave amplitude to tissue Doppler E′ wave amplitude (E/E′) ratio,

the flow Doppler E wave amplitude to tissue Doppler A wave amplitude (E/A) ratio, LV vol; d, LV vol; s, and left ventricular stroke volume (LV stroke vol) of male WT and MW mice at 3 to 12 months (n = 10). Two-tailed nonparametric Mann–Whitney test (G) or unpaired Student's t test (G, H) was used. The number of samples in each group is indicated by n. The data are presented as means ± SEMs. ns, not significant.

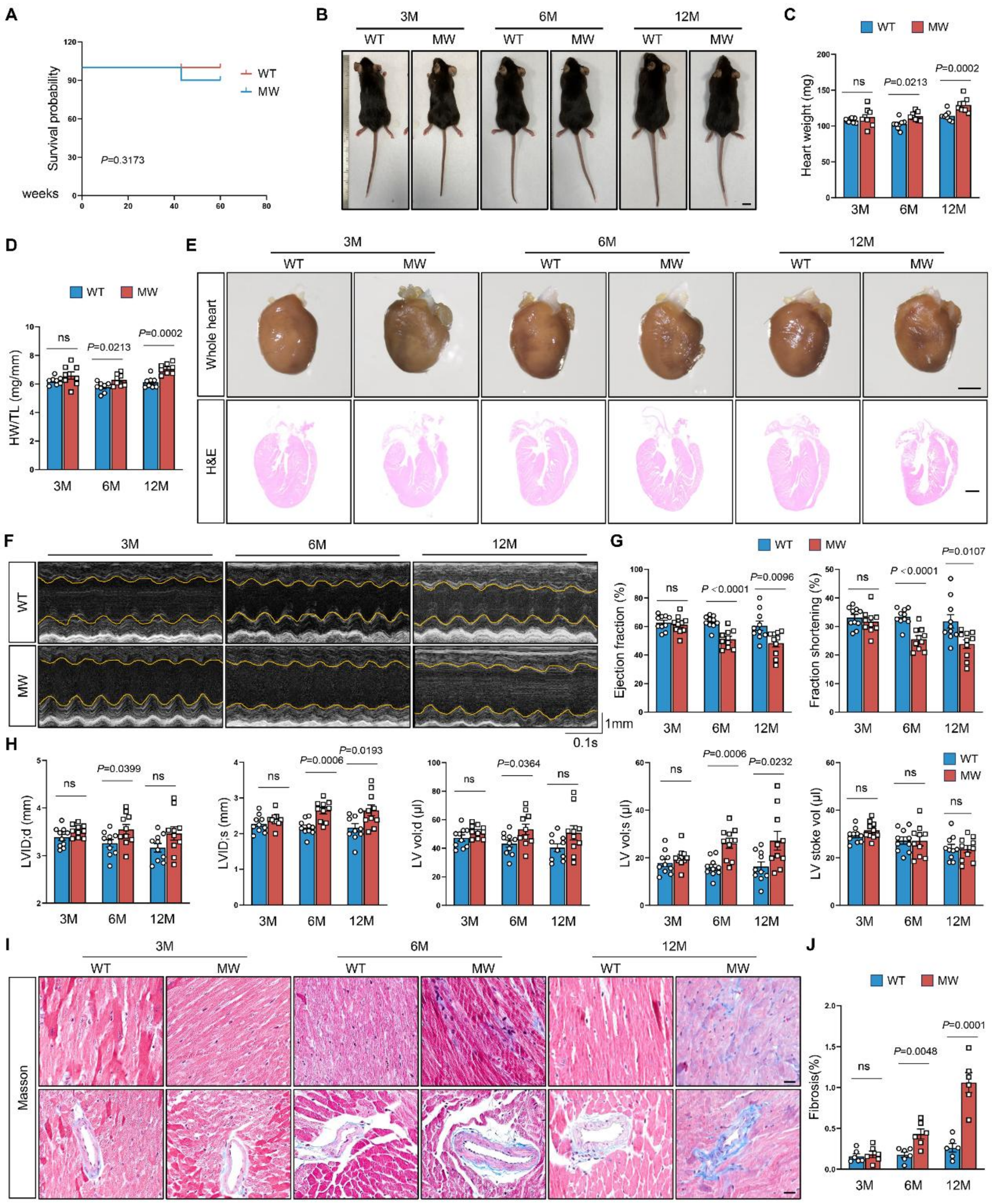

Figure S3. Female Piezo1^{D674Y} mice develop heart failure

A, Survival curves of female WT and MW mice (n = 10). The Kaplan–Meier method was used to compare the data via the log-rank test. **B**, Photograph of female WT and MW mice at 3 to 12 months (scale bar = 1 cm). **C**, Quantification of the HW of female WT and MW mice at 3 to 12 months (n = 8). **D**, HW/TL ratios of female WT and MW mice at 3 to 12 months (n = 8). **E**, Representative images of whole hearts (scale bar =

2 mm) and H&E-stained longitudinal heart sections (scale bar = 1 mm) from female WT and MW mice at 3 to 12 months. **F**, Representative M-mode echocardiographic images of female WT and MW mice at 3 to 12 months. **G**, Echocardiographic analysis of the EF and FS of female WT and MW mice at 3 to 12 months (n = 10). **H**, Echocardiographic analysis of the LVID; d, LVID; s, left ventricular end-diastolic volume (LV vol; d), left ventricular end-systolic volume (LV vol; s) LV stoke vol of female WT and MW mice at 3 to 12 months (n = 10). **I**, Representative images of Masson's trichrome (scale bar = 20 μm) staining of cardiac tissues from female WT and MW mice at 3 to 12 months. **J**, Quantification of the cardiac fibrosis area via Masson's trichrome staining in female WT and MW mice at 3 to 12 months of age (n = 6). Two-tailed unpaired Welch test (D) or Student's t test (C, D, G, H, J) was used. The number of samples in each group is indicated by n. The data are presented as means ± SEMs. ns, not significant.

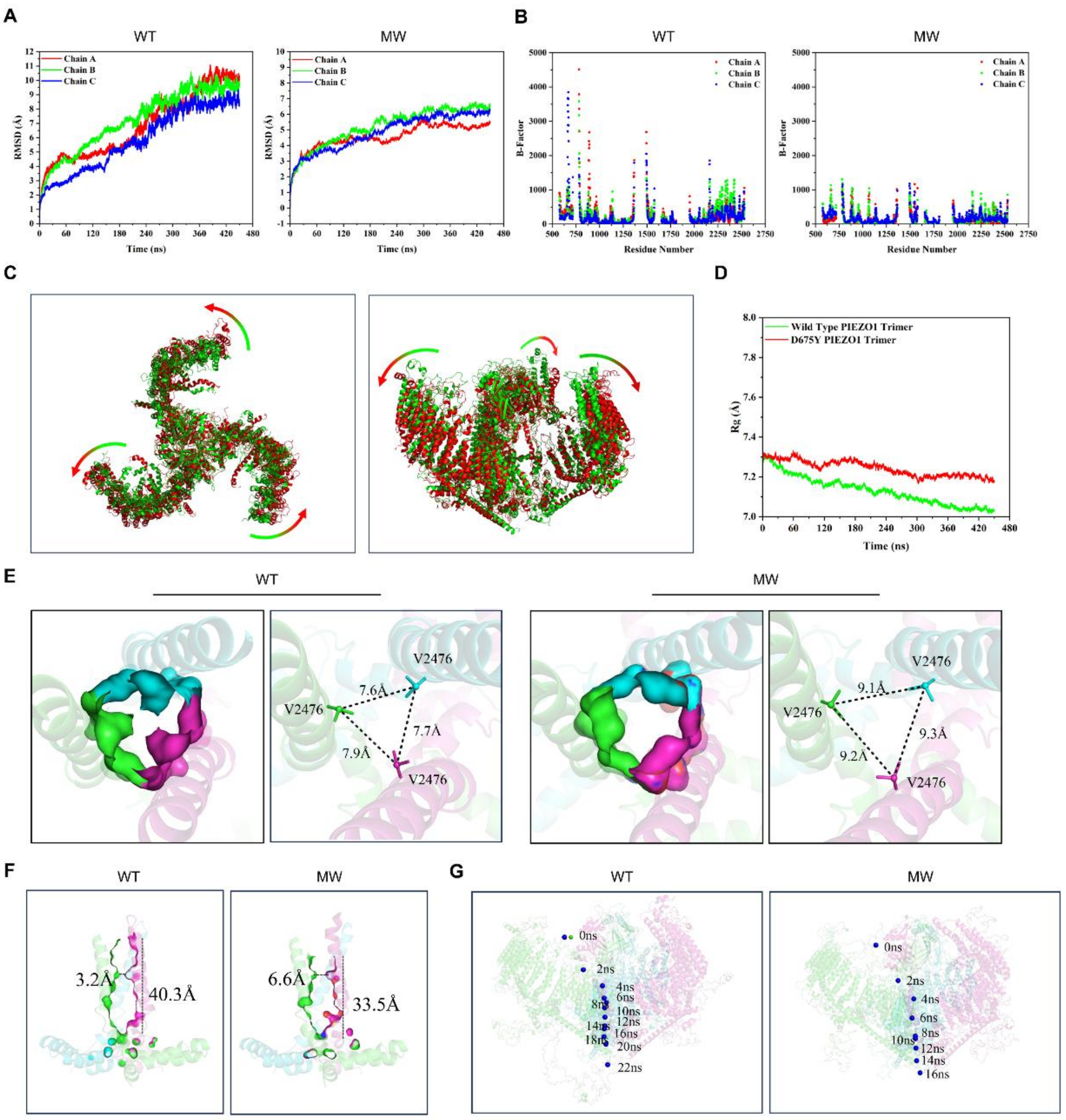

Figure S4. Molecular simulation of WT Piezo1 and the Piezo1^{D674Y} mutant

A, RMSD of the WT and MW trimers during 450 ns MD simulations. **B**, Root-mean-square-fluctuation (RMSF/B-factor ratio) of residues of the WT and MW trimers during the equilibrium stages of the 450 ns MD simulations. **C**, Top view and side view of the overlap of the WT and MW trimers after 450 ns MD simulations. **D**, The Rg of the WT and MW trimers during 450 ns MD simulations. **E**, The ion channel region of the WT and MW trimers. **F**, The ion channel region and bottleneck area of the WT and MW trimers. **G**, Analysis of the transit of Ca^{2+} ions through the WT and MW trimers.

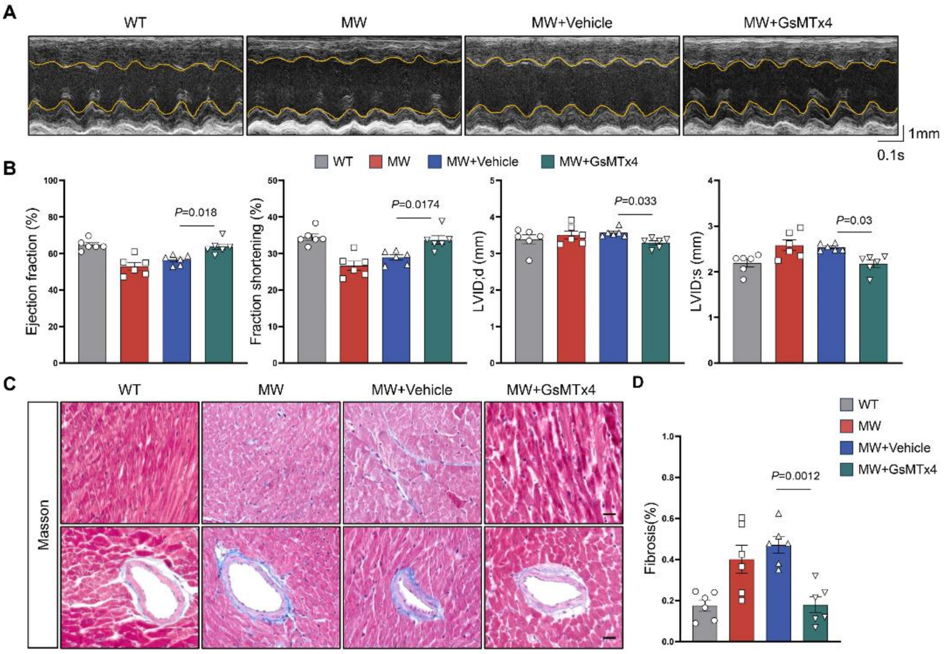

Figure S5. Pharmacological inhibition of PIEZO1 ameliorates cardiac injury in mutant mice

A, Representative M-mode echocardiographic images of male WT and MW mice treated with vehicle or GsMTx4 at 3 months. **B**, Echocardiographic analysis of the EF, FS, LVID; d, and LVID; s in male WT mice and MW mice treated with vehicle or GsMTx4 at 3months (n = 6). **C**, Representative images of Masson's trichrome (scale bar=20 μm) staining of cardiac tissues from male WT and MW mice treated with vehicle or GsMTx4 at 3 months. **D**, Quantification of the area of cardiac fibrosis via Masson's trichrome staining in male WT and MW mice treated with vehicle or GsMTx4 at 3 months (n = 6). One-way ANOVA with Tukey's test (B, D) or the Kruskal–Wallis's test (B) was used for the comparison of multiple groups. The number of samples in each group is indicated by n. The data are presented as means ± SEMs. ns, not significant.

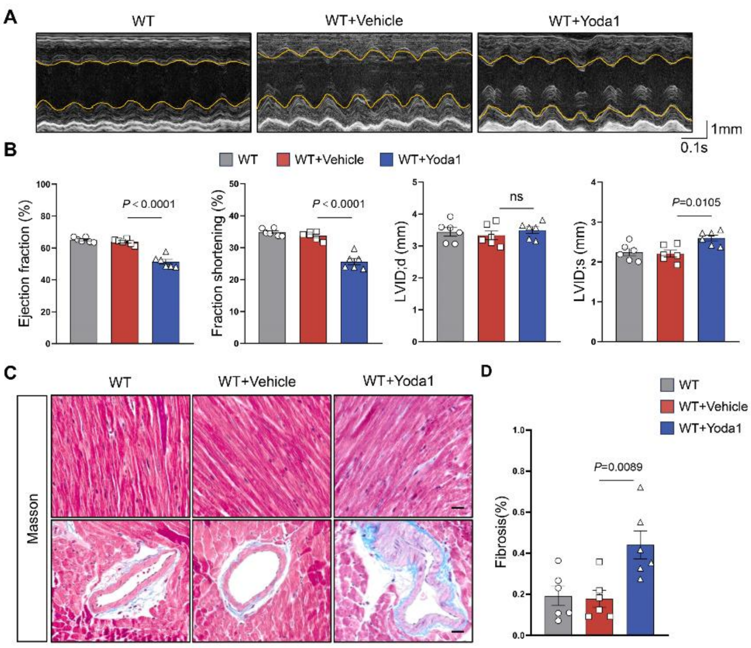

Figure S6. Pharmacological activation of PIEZO1 results in cardiac injury

A, Representative M-mode echocardiographic images of male WT mice treated with vehicle or Yoda1 at 3 months. **B,** Echocardiographic analysis of the EF, FS, LVID; d, and LVID; s in male WT and MW mice treated with vehicle or GsMTx4 at 3 months (n = 6). One-way **C**, Representative images of Masson's trichrome (scale bar=20 μm) staining of cardiac tissues from male WT mice treated with vehicle or Yoda1 at 3 months. **D**, Quantification of the area of cardiac fibrosis via Masson's trichrome staining in male WT mice treated with vehicle or Yoda1 at 3 months (n = 6). One-way ANOVA with Tukey's test was used for the comparison of multiple groups (B, D). The number of samples in each group is indicated by n. The data are presented as means ± SEMs. ns, not significant.

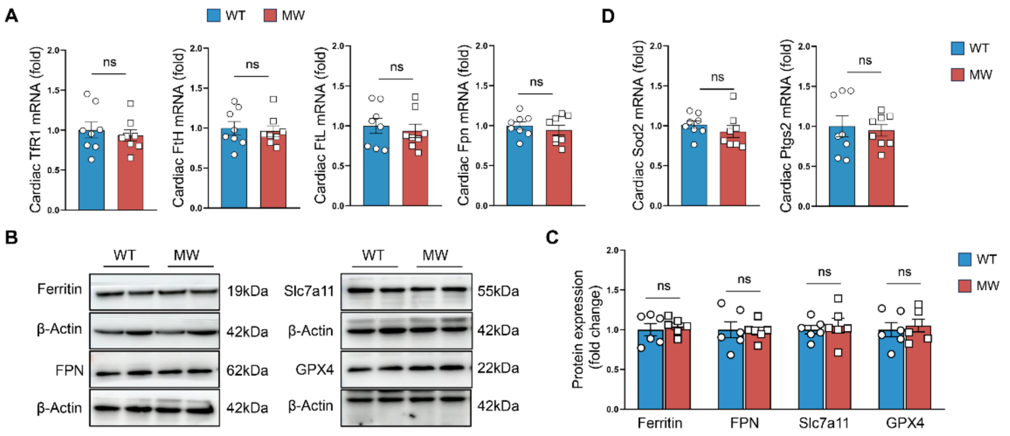

Figure S7. The Piezo1 GOF mutation impairs cardiac function without altering iron homeostasis

A, Relative levels of cardiac TfR1, FtH, FtL, and Fpn mRNA in male WT and MW mice at 3 months (n = 8). **B-C**, Representative western blot images (B) and quantitative analyse (C) of ferritin, FPN, Slc7a11 and GPX4 in cardiac tissues from male WT and MW mice at 3 months (n = 6). **D**, Relative levels of cardiac Sod2 and Ptgs2 mRNA in male WT and MW mice at 3 months (n = 8). Two-tailed unpaired Student's t test was used (A, C, D). The number of samples in each group is indicated by n. The data are presented as means ± SEMs. ns, not significant.

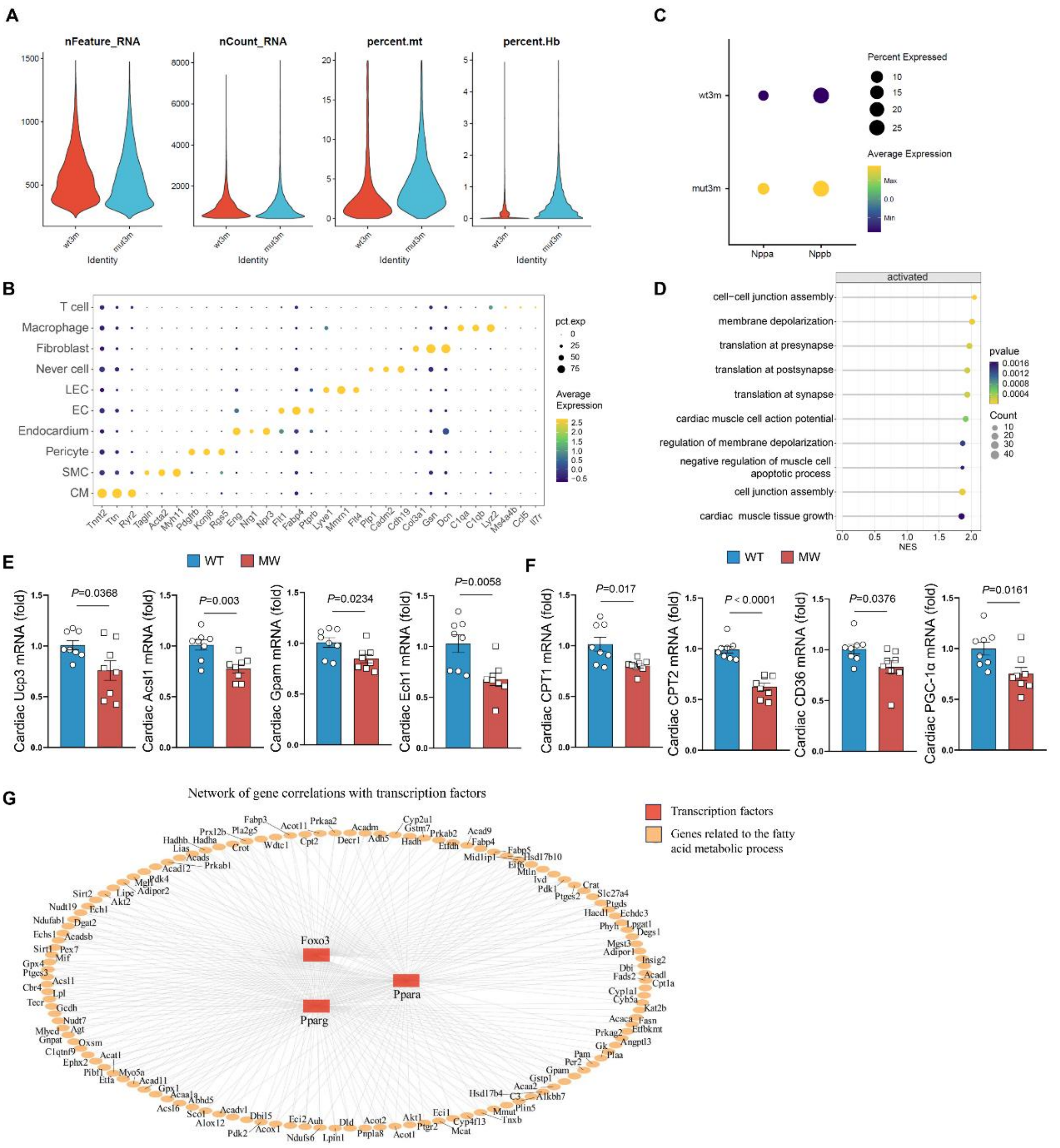

Figure S8. scRNA-seq of hearts from mice with Piezo1 GOF mutation

A, Violin plots illustrating quality control metrics across groups: nFeature_RNA (number of detected features), nCount_RNA (total RNA count), percent.mt (percentage of mitochondrial genes), and percent.Hb (percentage of hemoglobin genes). **B**, Dot plot showing the expression levels of marker genes across cell types, with the dot color representing the average expression level and the dot size indicating the percentage of each cell type expressing the gene. **C**, Dot plot illustrating the expression of Nppa and Nppb in male WT and MW mice at 3 months. **D**, Dot plot depicting GSEA-based

pathway enrichment in male MW mice relative to male WT at 3 months. *P* values were determined by GSEA using permutation tests and adjusted using the BH correction method. **E**, Relative mRNA levels of cardiac Ucp3, Acsl1, Gpam and Ech1 in male WT and MW mice at 3 months (n = 8). **F**, Relative mRNA levels of cardiac CPT1, CPT2, CD36 and PGC-1α in male WT and MW mice at 3 months (n = 8). **G**, Network of gene correlations with TFs. Two-tailed unpaired Student's t test was used (E, F). The number of samples in each group is indicated by n. The data are presented as means ± SEMs. ns, not significant.

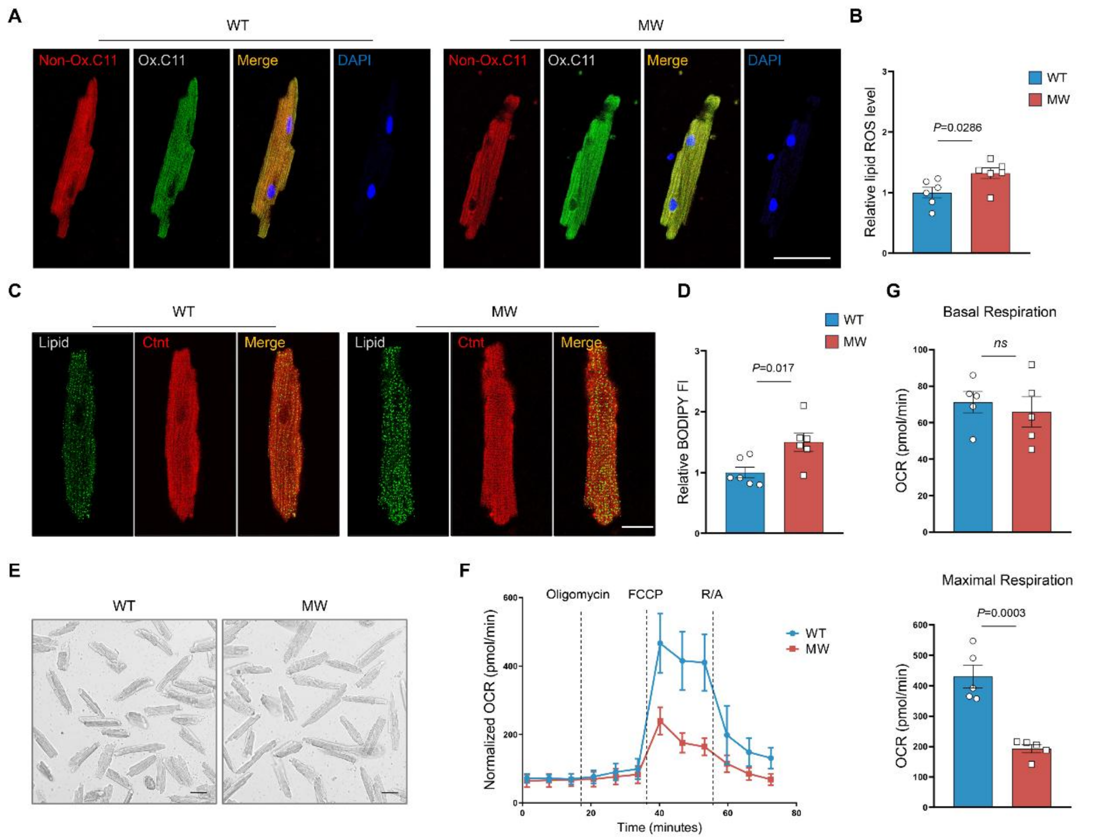

Figure S9. PIEZO1 GOF impairs lipid metabolism in cardiomyocytes

A-B, Representative images (A) and quantitative analysis (B) of C11-BODIPY-stained cardiomyocytes (scale bar = 20μm) from male WT and MW mice at 3 months (n = 6). **C-D**, Representative images (C) and quantitative analysis (D) of BODIPY-stained cardiomyocytes (scale bar = 20 μm) from male WT and MW mice at 3 months (n = 6). **E**. The morphology of cardiomyocytes (scale bar = 20 μm) isolated from male WT and MW mice at 3 months. **F**, The OCRs of cardiomyocytes from male WT and MW mice at 3 months. **G**, Quantitative analysis of basal respiration and maximal respiration in cardiomyocytes from male WT and MW mice at 3 months (n = 5). Two-tailed unpaired Student’s t test was used (B, D, G). The number of samples in each group is indicated by n. The data are presented as means ± SEMs. ns, not significant.

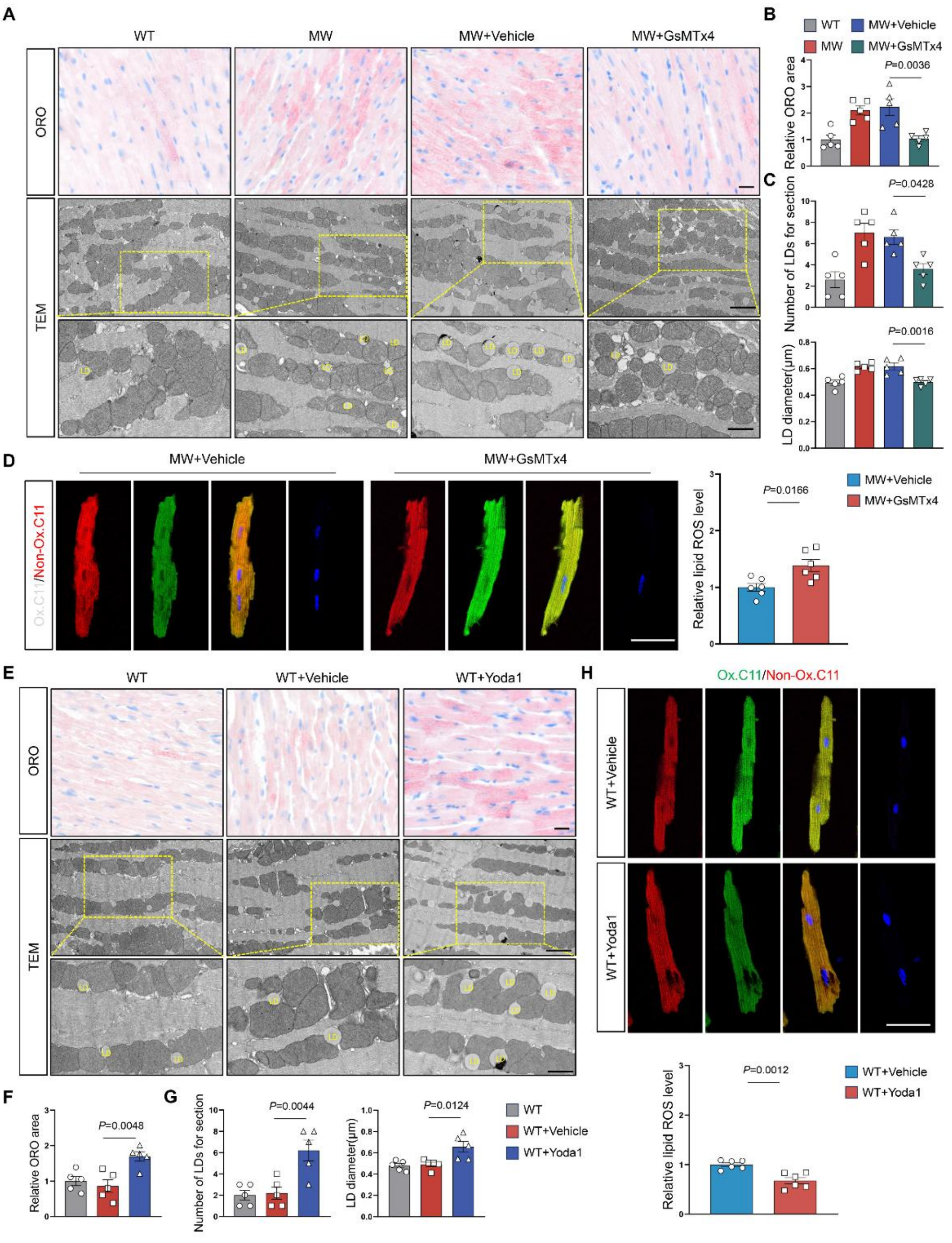

Figure S10. Pharmacological modulation of PIEZO1 channel activity influences cardiac lipid metabolism

A, Representative images of myocardial ORO (scale bar = 20 μm) staining and TEM images (scale bar = 2 μm and 1 μm) from male WT and MW mice treated with vehicle or GsMTx4 for 3 months. **B**, Quantification of ORO staining in male WT and MW mice treated with vehicle or GsMTx4 for 3 months (n = 5). **C**, Quantification of the LD

number and diameter in male WT and MW mice treated with vehicle or GsMTx4 at 3 months (n = 5). **D**, Representative images and quantitative analysis of C11-BODIPY-stained cardiomyocytes (scale bar = 20 μm) from male MW mice treated with vehicle or GsMTx4 at 3 months (n = 6). **E**, Representative images of myocardial ORO (scale bar = 20 μm) staining and TEM images (scale bar = 2 μm and 1 μm) from male WT mice treated with vehicle or Yoda1 at 3 months. **F,** Quantification of ORO staining in male WT mice treated with vehicle or Yoda1 at 3 months (n = 5). **G**, Quantification of the LD number and diameter in male WT mice treated with vehicle or Yoda1 at 3 months (n = 5). **H**, Representative images and quantitative analysis of C11-BODIPY-stained cardiomyocytes (scale bar = 20 μm) from male WT mice treated with vehicle or Yoda1 at 3 months (n = 6). One-way ANOVA with Tukey's test was used for the comparison of multiple groups (B, C, F, G). Two-tailed unpaired Student's t test was used (D, H). The number of samples in each group is indicated by n. The data are presented as means ± SEMs. ns, not significant.

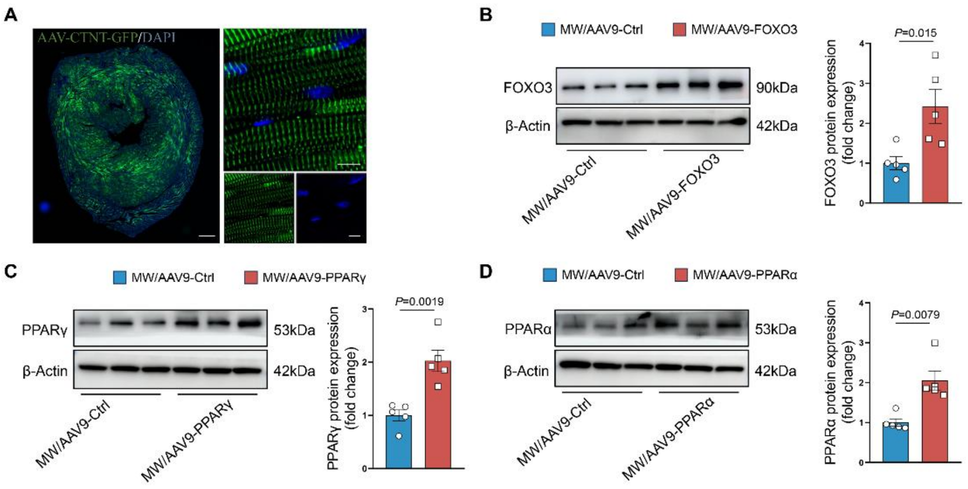

Figure S11. AAV9-mediated gene delivery system

A, Representative images of mouse hearts infected with AAV-CTNT-GFP (scale bar = 500 μm and 8 μm). **B**, Representative western blot images and quantitative analyses of FOXO3 expression in cardiac tissues from male MW mice injected with the indicated AAV9-FOXO3 vector (n = 5). **C**, Representative western blot images and quantitative analyses of PPARγ expression in cardiac tissues from male MW mice injected with the indicated AAV9-PPARγ vector (n = 5). **D**, Representative western blot images and quantitative analyses of PPARα expression in cardiac tissues from male MW mice injected with the indicated AAV9- PPARα vector (n = 5). Two-tailed unpaired Student's t test was used (B, C, D). The number of samples in each group is indicated by n. The data are presented as means ± SEMs. ns, not significant.

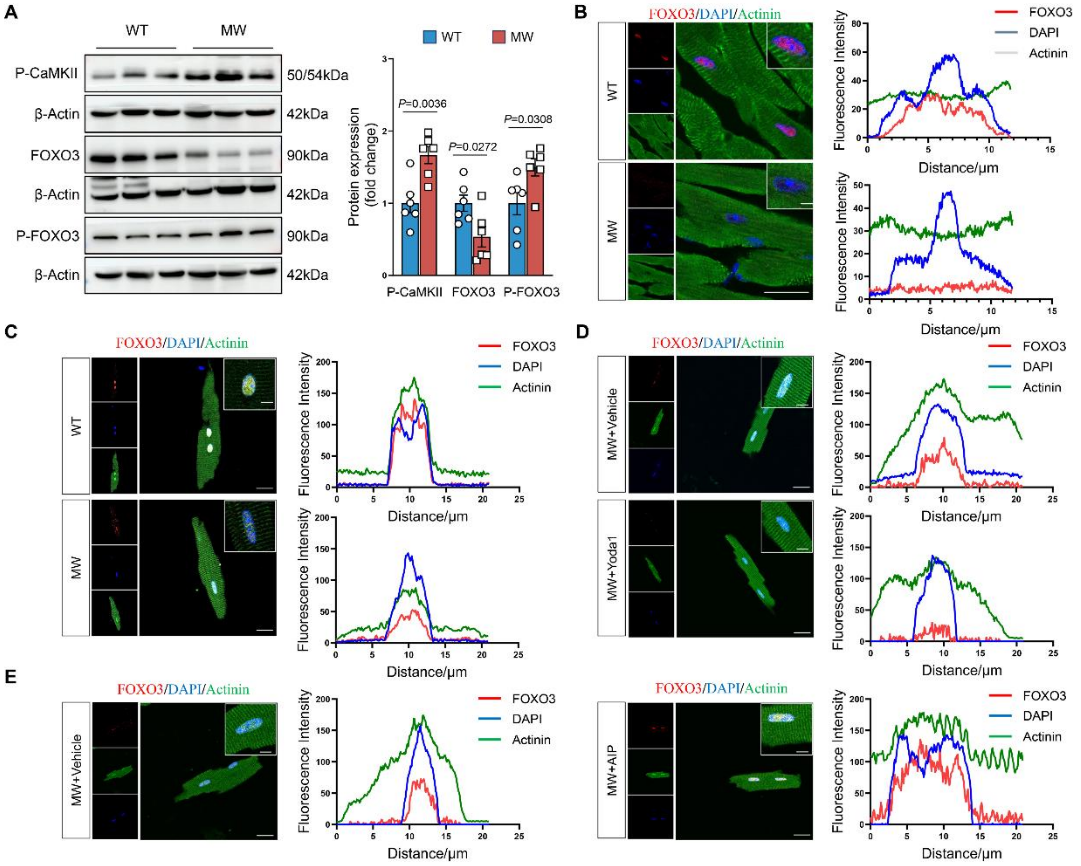

Figure S12. The CaMKII/FOXO3 signaling axis functions as a downstream mediator of PIEZO1

A, Representative western blot images and quantitative analyses of P-CaMKII, FOXO3 and P-FOXO3 levels in cardiac tissues from male WT and MW mice (n = 6). **B**, Representative images of FOXO3 localization and FOXO3 intensity traces (scale bar = 20 μm and 5 μm) in cardiac tissues from male WT and MW mice. **C**, Representative images of FOXO3 localization and FOXO3 intensity traces (scale bar = 20 μm and 5 μm) in cardiomyocytes from male WT and MW mice. **D**, Representative images of FOXO3 localization and FOXO3 intensity traces (scale bar = 20 μm and 5 μm) in cardiomyocytes treated with Yoda1 from male MW mice. **E**, Representative images of FOXO3 localization and FOXO3 intensity traces (scale bar = 20 μm and 5 μm) in cardiomyocytes treated with AIP from male MW mice. Two-tailed unpaired Student's t

test was used (A). The number of samples in each group is indicated by n. The data are presented as means ± SEMs. ns, not significant.

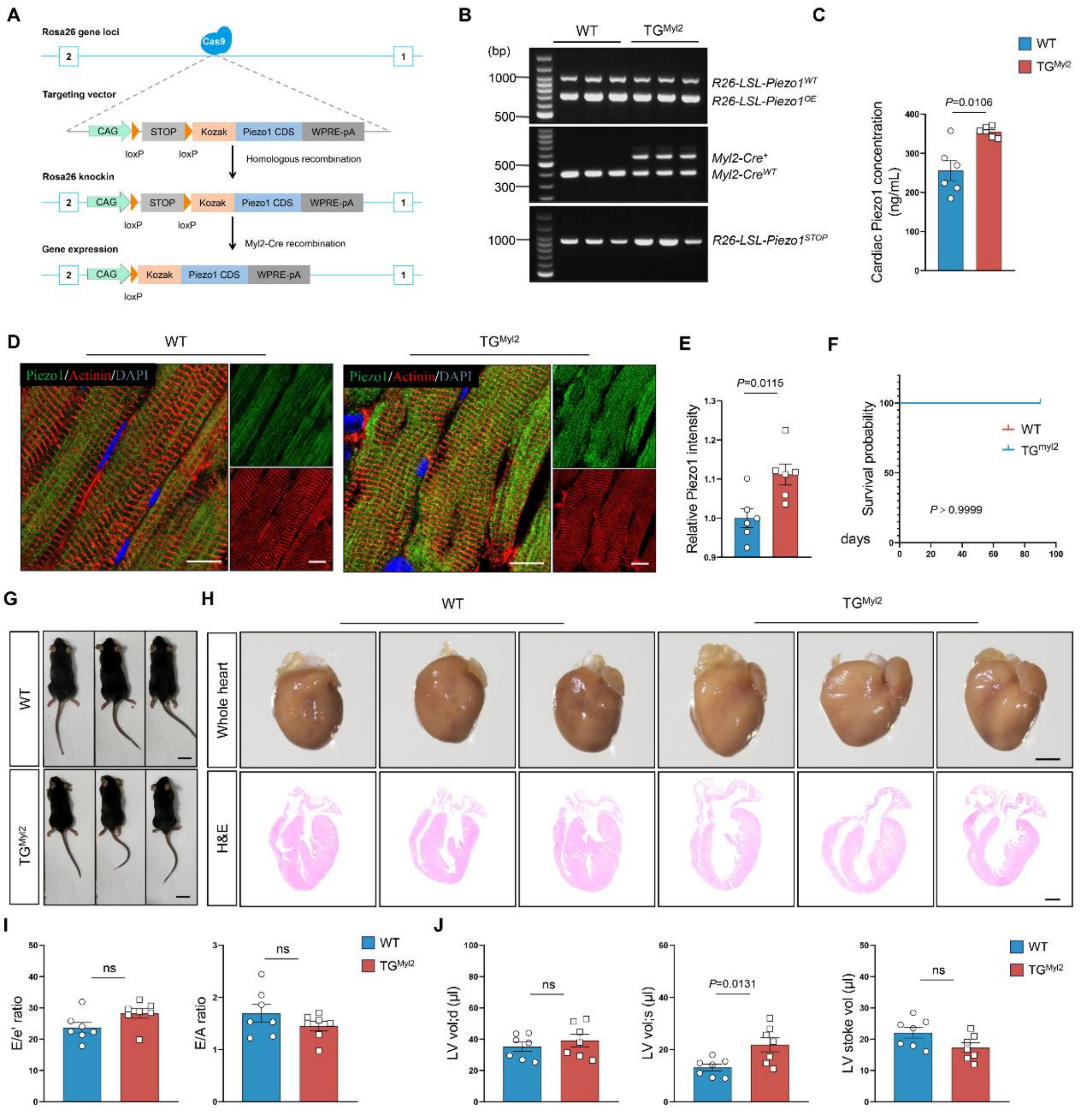

Figure S13. Generation and validation of *Piezo1*-TG^{Myl2} mice

A, Diagram of the generation of conventional Piezo1-overexpressing mice. **B**, Representative image of PCR genotyping of wild-type (WT) and *Piezo1*-TG^{Myl2} (TG^{Myl2}) mice. **C**, Quantification of the cardiac piezo1 concentration in cardiac tissues from WT and TG^{Myl2} mice (n = 6). **D**, Representative immunofluorescence images of heart sections from WT and TG^{Myl2} mice validating the overexpression of Piezo1 in cardiomyocytes. **E**, Quantification of cardiac Piezo1 levels in cardiac tissues from WT and TG^{Myl2} mice (n = 6). **F**, Survival curves of male WT and TG^{Myl2} mice (n = 10). The Kaplan–Meier method was used to compare the data via the log-rank test. **G**,

Photograph of male WT and TG^{Myl2} mice at 6 weeks (scale bar = 2 cm). **H**, Representative images of whole hearts (scale bar = 2 mm) and H&E-stained longitudinal heart sections (scale bar = 1 mm) from male WT and TG^{Myl2} mice at 6 weeks. **I**, Echocardiographic analysis of E/eʹratio and E/A ratio in male WT and TG^{Myl2} mice at 6 weeks (n = 7). **J**, Echocardiographic analysis of the LV vol; d, LV vol; s, and LV stoke vol in male WT and TG^{Myl2} mice at 6 weeks (n = 7). The two-tailed unpaired Welch test (C) or Student's t test (E, I, J) was used. The number of samples in each group is indicated by n. The data are presented as means ± SEMs. ns, not significant.

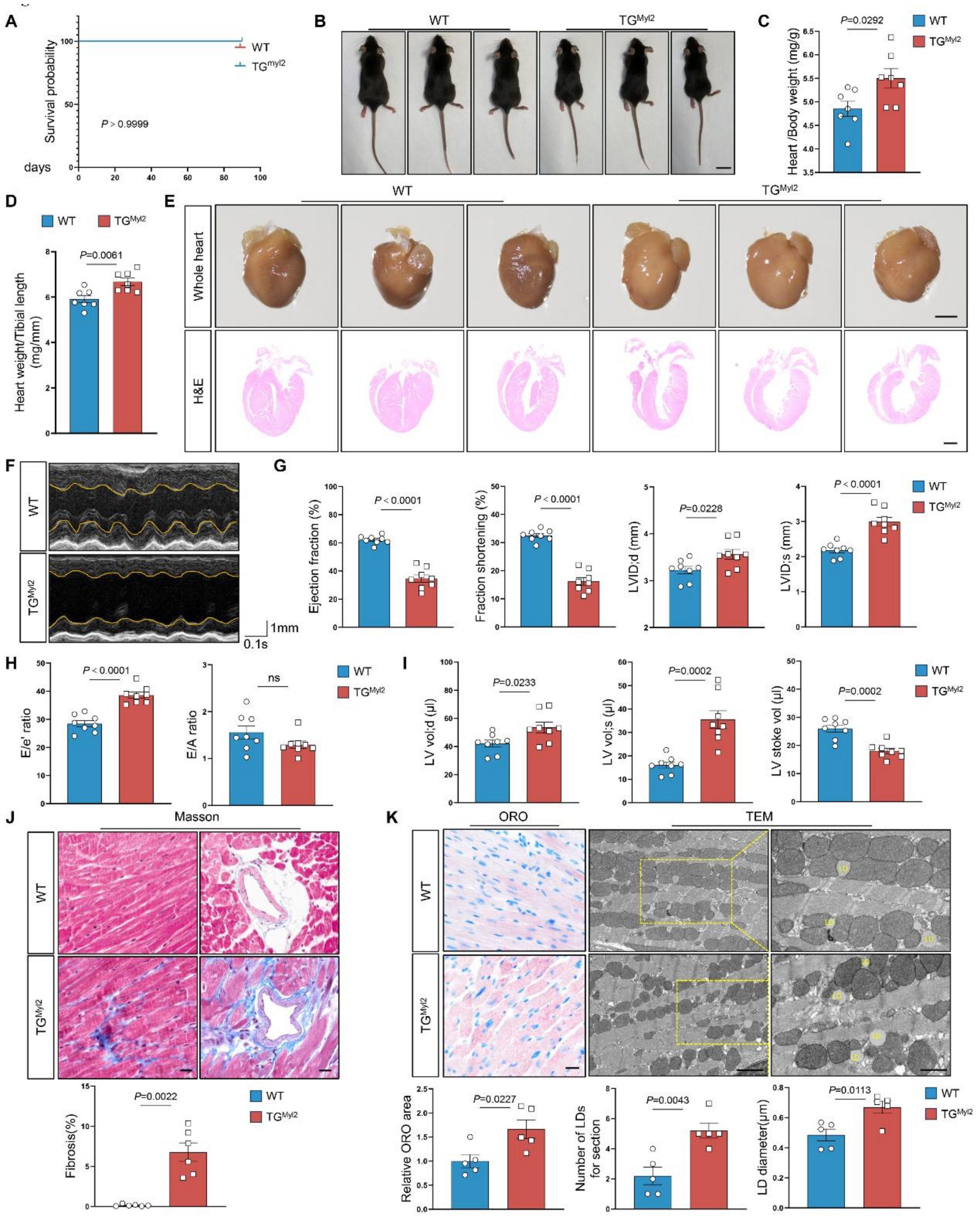

Figure S14. Female mice with cardiac-specific overexpression of Piezo1 also develop severe cardiac damage and cardiolipotoxicity

A, Survival curves of female WT and TG^{Myl2} mice (n = 10). The Kaplan–Meier method was used to compare the data via the log-rank test. **B**, Photograph of female WT and TG^{Myl2} mice at 6 weeks (scale bar = 2 cm). **C-D**, HW/BW ratio (C) and HW/TL ratio (D) of female WT and TG^{Myl2} mice at 6 weeks (n = 7). **E**, Representative images of

whole hearts (scale bar = 2 mm) and H&E-stained longitudinal heart sections (scale bar = 1 mm) from female WT and TG^{Myl2} mice at 6 weeks. **F**, Representative M-mode echocardiographic images of female WT and TG^{Myl2} mice at 6 weeks. **G-I,** Echocardiographic analysis of the EF (G), FS (G), LVID; d (G), LVID; s (G), E/e′ratio (H), E/A ratio (H), LV vol; d (I), LV vol; s (I), and LV stoke vol (I)in female WT and TG^{Myl2} mice at 6 weeks (n = 8). **J**, Representative images and quantitative analyses of Masson's trichrome (scale bar=20 μm) staining of cardiac tissues from female WT and TG^{Myl2} mice at 6 weeks (n = 6). **K**, Representative images and quantitative analyses of ORO staining (scale bar = 20 μm) and TEM (scale bar = 2 μm and 1 μm) from female WT and TG^{Myl2} mice at 6 weeks (n = 5). The two-tailed unpaired Student's t test (C, D, G, H, I, K), unpaired Welch test (G, J), or nonparametric Mann–Whitney test (H) was used. The number of samples in each group is indicated by n. The data are presented as means ± SEMs. ns, not significant.

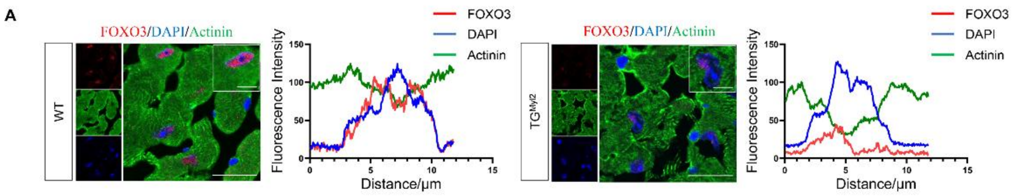

Figure S15. The nuclear localization of FOXO3 in *Piezo1*-TG^{Myl2} mice

A, Representative images of FOXO3 localization and FOXO3 intensity traces (scale bar = 20 μm and 5 μm) in cardiac tissues from male WT and TG^{Myl2} mice.

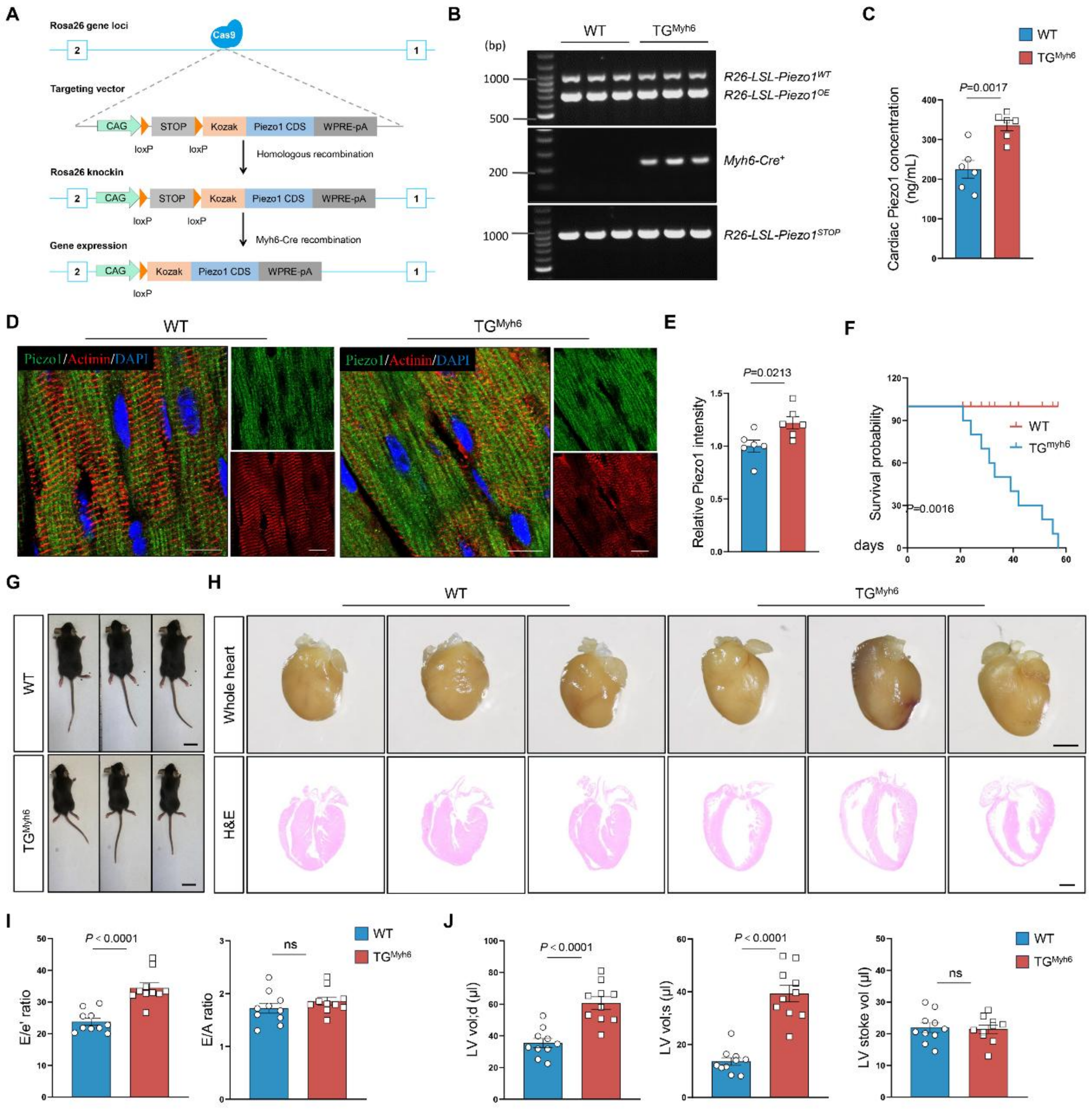

Figure S16. Generation and characterization of Piezo1-TG^{Myh6} mice

A, Diagram of the generation of conventional Piezo1-overexpressing mice. **B**, Representative image of PCR genotyping of WT and TG^{Myh6} mice. **C**, Quantification of cardiac Piezo1 concentrations in cardiac tissues from WT and TG^{Myh6} mice (n = 6). **D**, Representative immunofluorescence images of heart sections from WT and TG^{Myh6} mice validating the overexpression of Piezo1 in cardiomyocytes. **E**, Quantification of cardiac Piezo1 levels in cardiac tissues from WT and TG^{Myh6} mice (n = 6). **F**, Survival curves of male WT and TG^{Myh6} mice (n = 10). The Kaplan–Meier method was used to compare the data via the log-rank test. **G**, Photograph of male WT and TG^{Myh6} mice at 4 weeks (scale bar = 2 cm). **H**, Representative images of whole hearts (scale bar = 2

mm) and H&E-stained longitudinal heart sections (scale bar = 1 mm) from male WT and TG^{Myh6} mice at 4 weeks. **I**, Echocardiographic analysis of the E/e′ ratio and E/A ratio in male WT and TG^{Myh6} mice at 4 weeks (n = 10). **J**, Echocardiographic analysis of the LV vol; d, LV vol; s, and LV stoke vol in male WT and TG^{Myh6} mice at 4 weeks (n = 10). Two-tailed unpaired Student's t test was used (C, E, I, J). The number of samples in each group is indicated by n. The data are presented as means ± SEMs. ns, not significant.

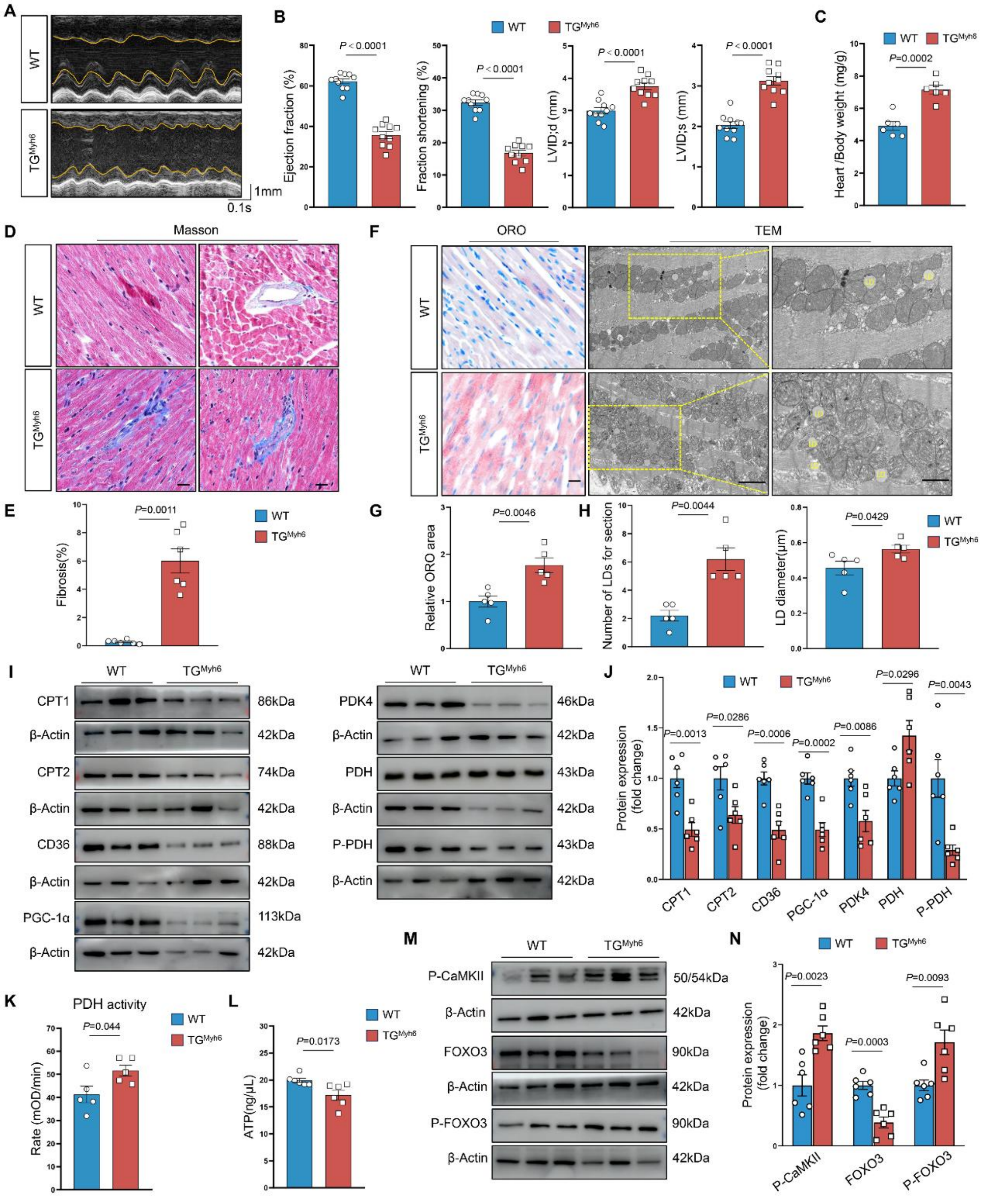

Figure S17. Cardiomyocyte-specific Piezo1 overexpression impairs cardiac function and cardiac lipid metabolism

A, Representative M-mode echocardiographic images of male WT and *Piezo1*-TG^{Myh6} (TG^{Myh6}) mice at 4 weeks. **B,** Echocardiographic analysis of the EF, FS, LVID;d and LVID;s in male WT and TG^{Myh6} mice at 4 weeks (n = 10). **C,** HW/BW ratios of male WT and TG^{Myh6} mice at 4 weeks (n = 6). **D,** Representative images of Masson's

trichrome (scale bar=20 μm) staining of cardiac tissues from male WT and TG^{Myh6} mice at 4 weeks. **E**, Quantification of the cardiac fibrosis area via Masson's trichrome staining in male WT and TG^{Myh6} mice at 4 weeks (n = 6). **F**, Representative images of ORO (scale bar = 20 μm) staining and TEM of cardiac tissues from male WT and TG^{Myh6} mice at 4 weeks. **G**, Quantification of ORO staining in male WT and TG^{Myh6} mice at 4 weeks (n = 5). **H**, Quantification of the LD number and diameter in male WT and TG^{Myh6} mice at 4 weeks (n = 5). **I-J**, Representative western blot images (I) and quantitative analyses (J) of CPT1, CPT2, CD36, PGC-1α, PDK4, PDH and P-PDH in cardiac tissues from male WT and TG^{Myh6} mice (n = 6). **K-L**, PDH activity (K) and concentrations of ATP (L) in cardiac tissues from male WT and TG^{Myh6} mice at 4 weeks (n = 5, 6). **M-N**, Representative western blot images (M) and quantitative analyses (N) of P-CaMKII, FOXO3 and P-FOXO3 in cardiac tissues from male WT and TG^{Myh6} mice (n =6). Two-tailed unpaired Student's t test was used (B, C, E, G, H, J, K, L, N). The number of samples in each group is indicated by n. The data are presented as means ± SEMs. ns, not significant.

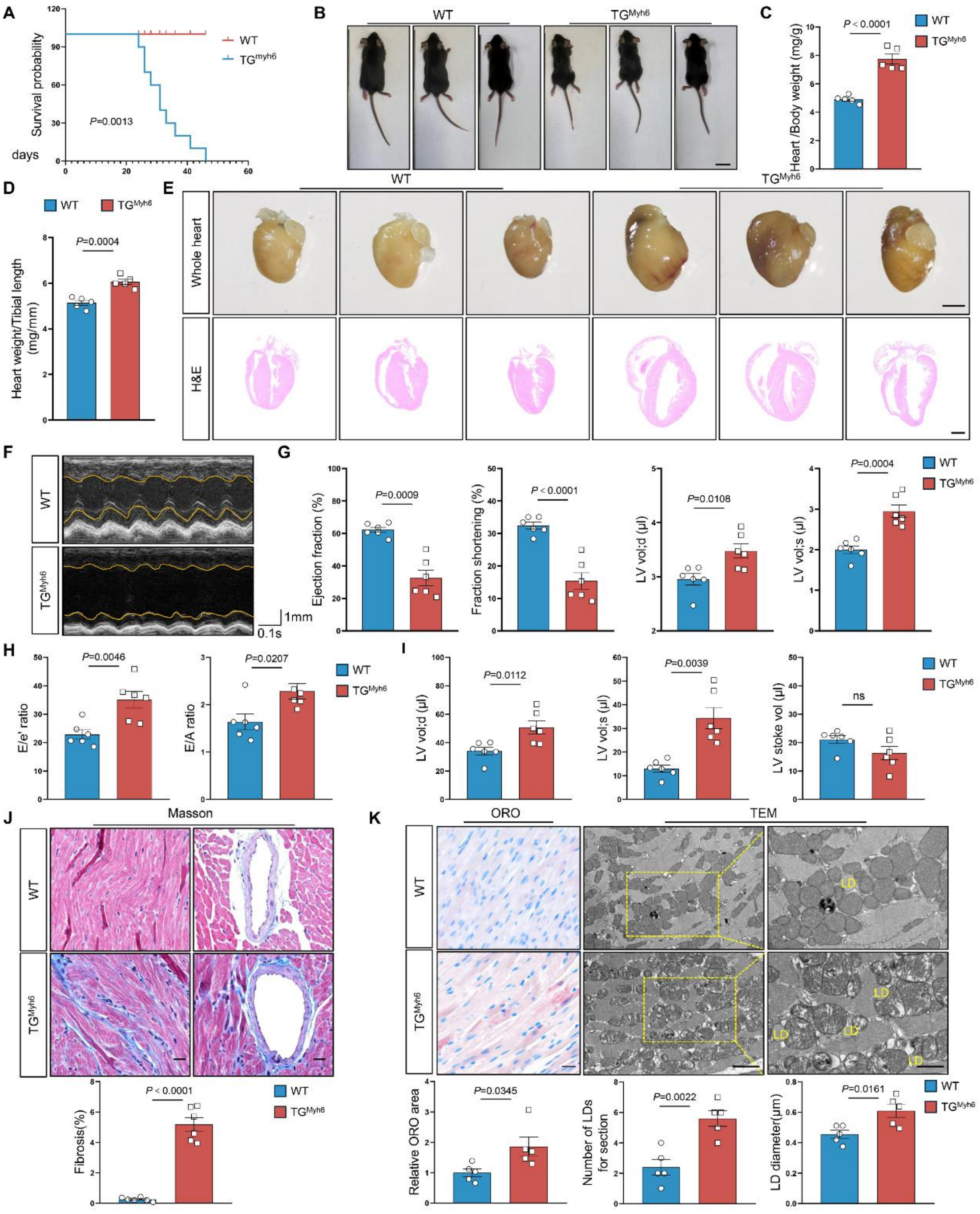

Figure S18. Cardiac-specific overexpression of Piezo1 induces cardiomyopathy and cardiolipotoxicity in female mice

A, Survival curves of female WT and TG^{Myh6} mice (n = 10). The Kaplan–Meier method was used to compare the data via the log-rank test. **B**, Photograph of female WT and TG^{Myh6} mice at 4 weeks (scale bar = 2 cm). **C-D**, HW/BW ratio (C) and HW/TL ratios (D) of female WT and TG^{Myh6} mice at 4 weeks (n = 5). **E**, Representative images of

whole hearts (scale bar = 2 mm) and H&E-stained longitudinal heart sections (scale bar = 1 mm) from female WT and TG^{Myh6} mice at 4 weeks. **F**, Representative M-mode echocardiographic images of female WT and TG^{Myh6} mice at 4 weeks. **G-I,** Echocardiographic analysis of the EF (G), FS (G), LVID; d (G), LVID; s (G), E/e′ratio (H), E/A ratio (H), LV vol; d (I), LV vol; s (I), and LV stoke vol (I)in female WT and TG^{Myl2} mice at 6 weeks (n = 6). **J**, Representative images and quantitative analyses of Masson's trichrome (scale bar=20 μm) staining of cardiac tissues from female WT and TG^{Myh6} mice at 4 weeks (n = 6). **K**, Representative images and quantitative analyses of ORO staining (scale bar = 20 μm) and TEM (scale bar = 2 μm and 1 μm) from female WT and TG^{Myh6} mice at 4 weeks (n = 6). The two-tailed unpaired Student's t test(C, D, G, H, I, K), or Welch test (G, I, J) was used. The number of samples in each group is indicated by n. The data are presented as means ± SEMs. ns, not significant.

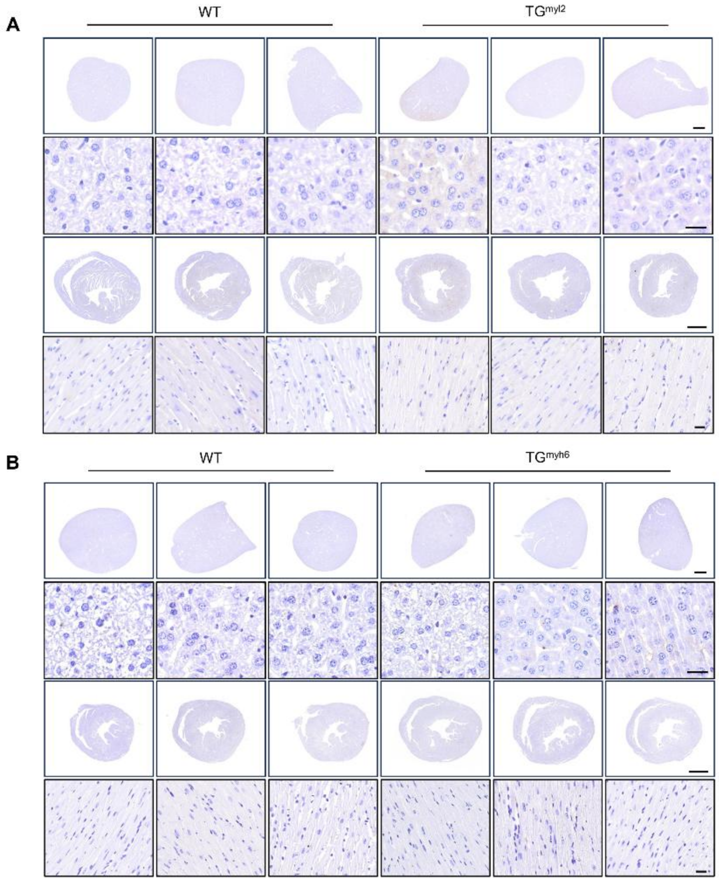

Figure S19. Cardiac-specific overexpression of Piezo1 impairs cardiac function without causing iron overload

A, Representative images of Perls Prussian blue staining (scale bar = 1 mm and 10 μm) of liver and cardiac tissues from male WT and TG^{Myh6} mice. **B**, Representative images of Perls Prussian blue staining (scale bar = 1 mm and 10 μm) of liver and cardiac tissues from male WT and TG^{Myh6} mice.

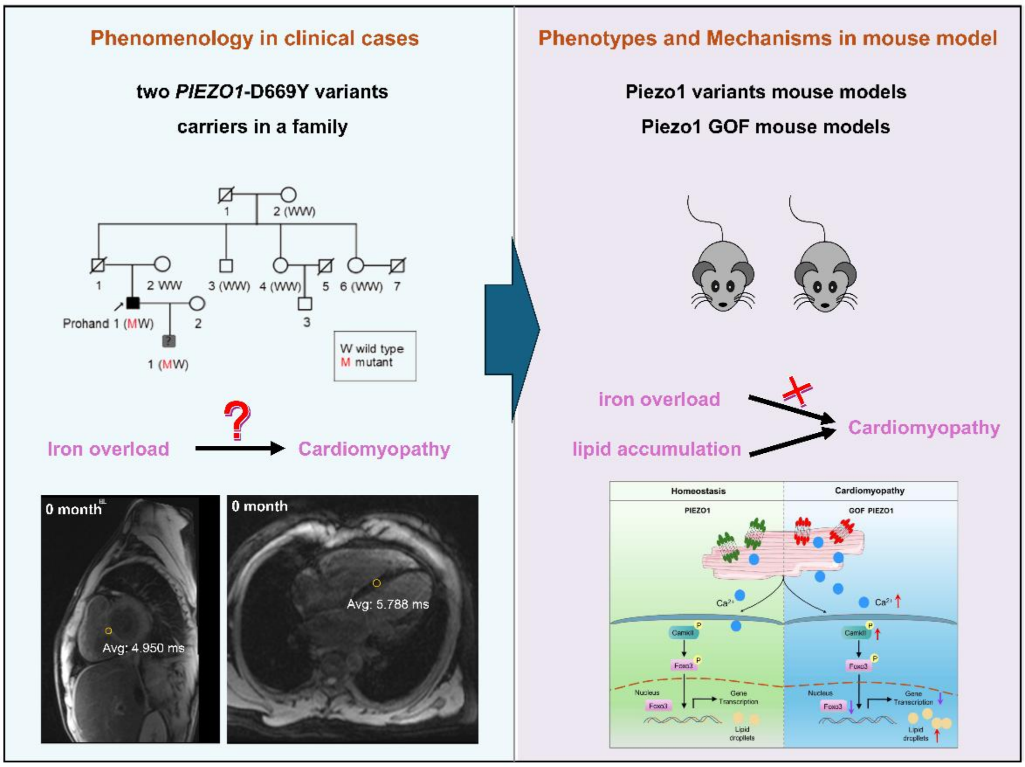

Figure S20. Graphical Abstract.

Schematic diagram showing the mechanism by which PIEZO1 gain-of-function mutation drives cardiomyopathy. PIEZO1 gain-of-Function mutation drives cardiomyopathy phenotype in human hearts and mouse model, largely independent of iron overload. PIEZO1 gain-of-Function mutation induces Intracellular Ca^{2+} overload and activation of the CaMKII, causing increased its phosphorylation of FOXO3 and nuclear translocation to affect its transcriptional activity, and thereby impairing lipid metabolism, resulting in impaired cardiac function and progression of cardiomyopathy.

Table S1. Longitudinal echocardiographic assessment of the proband with PIEZO1-D669Y variant (Father)

	Reference range	Baseline	Month 1	Month 8	Month 13	Month 26	Month 30	Month 38
LVEF (%)	52.1-74.5	30	37	55	53	62	59	64
E/e' ratio	0.0-10.0	22.17	18	11.9	8.6	7.9	8.3	6.6
LA anteroposterior diameter (mm)	23.7-37.3	48	47	39	36	35	38	35
LV end-diastolic diameter (mm)	39.4-54.0	58	56	53	46	46	50	45
RV diameter (mm)	14.2-28.8	31	30	25	26	10	24	23
RA long-axis diameter (mm)	34.4-52.4	54	53	47	43	43	43	42
RA transverse diameter (mm)	26.4-45.2	50	48	45	37	39	34	38
Interventricular septum (mm)	6.2-10.6	10	8	9	8	11	8	9
Left ventricle posterior wall (mm)	6.2-10.6	11	10	8	10	10	8	9

Table S2. Longitudinal blood examination of the proband with PIEZO1-D669Y variant (Father)

	Reference range	Baseline	Month 1	Month 8	Month 13	Month 26	Month 30	Month 38
Red blood cell count (*10^12/L)	4.3-5.8	2.55	3.11	9.35	7.67	3.19	2.64	3.1
Hemoglobin (g/L)	130.0-175.0	81	133	99	123	135	115	125
Mean corpuscular volume (f/L)	82.0-100.0	119.1	119.1	113.6	119.1	113.8	115.9	106.8
Mean corpuscular hemoglobin (pg)	27.0-34.0	41.1	41.1	42.8	43.6	42.3	43.6	40.3
Total bilirubin (u mol/L)	5.0-21.0	162	90.1	73.5	66	67.5	69.4	39.5
Direct bilirubin (u mol/L)	0.0-6.0	43.1	12.6	13.3	14.6	15.6	17.9	12.8
Indirect bilirubin (u mol/L)	2.0-15.0	119.1	77.5	60.2	51.4	51.9	51.5	26.7
Serum Ferritin (ng/ml)	3.0-400.0	1590	1759	374	691	957	810	162
NT-proBNP (pg/ml)	0.0-125.0	3458	967	176	34.66	-	32	38.7
Hemoglobin A1c (%)	4.0-6.0	4.70	-	4.9	5.3	6.5	5.0	4.8
Low-Density Lipoprotein Cholesterol (mmol/L)	1.0-3.4	1.25	-	-	-	-	-	0.98
High-Density Lipoprotein Cholesterol (mmol/L)	0.8-2.0	0.94	-	-	-	-	-	-
Triacylglycerides (mmol/L)	0.3-1.7	1.39	-	-	-	-	-	7.74
Creatinine (u mol/L)	62.0-115.0	41	-	-	50	-	-	51

Table S3. Echocardiographic parameters of the male offspring carrying PIEZO1-D669Y mutation

	Reference range	Patient value
LVEF（%）	52.1-74.5	70
E/e' ratio	5.0-8.0	6
LA anteroposterior diameter (mm)	14.2-28.8	22
LV end-diastolic diameter (mm)	23.7-37.3	32
RV diameter (mm)	15.0-25.0	18
RA long-axis diameter (mm)	28.0-38.0	30.0
RA transverse diameter (mm)	28.0-38.0	30.0
Interventricular septum（mm）	6.2-10.6	7.0
Left ventricle posterior wall（mm）	6.2-10.6	7.0

Table S4. Blood examination of the male offspring carrying PIEZO1-D669Y mutation

	Reference range	Patient value
Red blood cell count (*10^12/L)	4.2-5.7	3.43
Hemoglobin (g/L)	118-156	107.0
Mean corpuscular volume (f/L)	77-92	88.6
Mean corpuscular hemoglobin (pg)	25-34	31.2
Total bilirubin (u mol/L)	5.0-21.0	93.7
Direct bilirubin (u mol/L)	<6.0	6.0
Indirect bilirubin (u mol/L)	2.0-15.0	87.7
Serum Ferritin (ng/ml)	13-400	419
NT-proBNP (pg/ml)	$\leqslant 125$	124

Table S5. Echo parameters of male WT and MW

	WT3m (♂)	MW3m (♂)	WT6m (♂)	MW6m (♂)	WT12m (♂)	MW12m (♂)
Heart Rate (bpm)	488.03±28.70	505.16±25.75	521.97±11.55	537.63±7.49	446.17±20.23	483.69±19.19
Diameter;s (mm)	2.16±0.09	2.75±0.06	2.30±0.09	2.93±0.08	2.44±0.06	2.89±0.06
Diameter;d (mm)	3.22±0.13	3.75±0.07	3.42±0.08	3.84±0.06	3.45±0.07	3.65±0.07
Volume;s (µL)	16.01±1.54	28.43±1.55	18.51±1.70	33.34±2.08	21.19±1.30	32.12±1.73
Volume;d (µL)	42.46±4.06	60.49±2.61	48.56±2.79	63.75±2.31	49.32±2.47	56.39±2.42
Stroke Volume (µL)	26.46±2.61	32.06±1.56	30.06±1.43	30.42±1.40	28.13±1.44	24.27±0.76
Ejection Fraction (%)	62.26±1.05	53.07±1.35	62.45±1.76	47.96±2.06	57.08±1.20	43.27±0.81
Fractional Shortening (%)	32.71±0.78	26.82±0.84	32.97±1.19	23.81±1.23	29.25±0.79	20.85±0.44
Cardiac Output (mL/min)	13.15±1.70	15.94±0.64	15.64±0.71	16.38±0.86	12.49±0.79	11.67±0.44
LV Mass (mg)	88.61±6.63	117.97±9.59	99.71±7.30	120.56±6.43	81.63±2.26	110.70±6.20
LV Mass Corrected (mg)	70.89±5.30	94.37±7.67	79.77±5.84	96.45±5.15	65.31±1.81	88.56±4.96
LVAW;s (mm)	1.08±0.06	1.20±0.07	1.21±0.06	1.08±0.06	0.95±0.03	1.04±0.06
LVAW;d (mm)	0.87±0.05	0.96±0.06	0.93±0.06	0.88±0.05	0.77±0.02	0.92±0.05
LVPW;s (mm)	1.09±0.04	1.04±0.06	1.10±0.05	1.09±0.04	1.03±0.03	0.96±0.05
LVPW;d (mm)	0.80±0.04	0.75±0.05	0.77±0.05	0.83±0.03	0.69±0.03	0.79±0.04
MV E (mm/s)	580.44±48.50	755.07±37.91	654.40±29.48	710.79±57.40	607.05±42.10	739.51±30.18
MV A (mm/s)	430.64±41.91	600.67±42.48	461.63±30.77	443.78±39.31	502.06±31.47	510.18±39.93
E' (mm/s)	-26.20±2.41	-22.19±1.19	-26.98±2.44	-22.40±2.35	-21.07±5.63	-27.70±2.22
A' (mm/s)	-20.27±3.16	-20.54±2.06	-22.22±1.76	-22.97±2.20	-16.10±1.78	-18.20±1.07
MV E/E'	-22.39±0.92	-34.60±2.07	-25.32±1.52	-32.65±2.33	-20.23±4.67	-27.59±1.44
MV E/A	1.40±0.09	1.29±0.08	1.45±0.07	1.62±0.07	1.21±0.03	1.49±0.08
E'/A'	1.40±0.10	1.18±0.12	1.23±0.08	0.98±0.05	1.30±0.35	1.52±0.08
A'/E'	0.75±0.06	0.94±0.10	0.84±0.05	1.04±0.05	0.51±0.13	0.67±0.03

Table S6. Echo parameters of female WT and MW

	WT3m (♀)	MW3m (♀)	WT6m (♀)	MW6m (♀)	WT12m (♀)	MW12m (♀)
Heart Rate (bpm)	511.21±11.99	521.27±9.15	510.41±12.94	500.59±12.74	520.11±14.51	482.86±23.34
Diameter;s (mm)	2.27±0.08	2.39±0.06	2.17±0.07	2.64±0.09	2.17±0.11	2.65±0.15
Diameter;d (mm)	3.38±0.06	3.52±0.04	3.26±0.08	3.55±0.10	3.16±0.09	3.46±0.14
Volume;s (μL)	17.86±1.53	20.11±1.30	15.97±1.23	26.10±2.11	16.29±1.99	27.17±3.91
Volume;d (μL)	47.20±2.18	51.63±1.44	43.19±2.47	53.23±3.69	40.35±2.74	50.80±4.99
Stroke Volume (μL)	29.34±0.96	31.51±1.12	27.21±1.51	27.13±2.09	24.06±1.59	23.63±1.55
Ejection Fraction (%)	62.65±1.66	61.19±1.93	63.25±1.34	51.09±1.97	60.50±3.23	48.30±2.70
Fractional Shortening (%)	33.07±1.14	32.20±1.36	33.37±0.92	25.53±1.22	31.79±2.35	23.83±1.51
Cardiac Output (mL/min)	14.95±0.44	16.40±0.58	13.84±0.75	13.64±1.23	12.46±0.77	11.53±1.10
LV Mass (mg)	88.83±5.33	96.89±5.75	97.06±3.90	106.94±6.21	96.52±7.97	109.55±11.63
LV Mass Corrected (mg)	71.06±4.26	77.51±4.60	77.65±3.12	85.56±4.97	77.22±6.37	87.64±9.30
LVAW;s (mm)	1.07±0.05	1.16±0.07	1.19±0.05	1.15±0.05	1.16±0.09	1.06±0.09
LVAW;d (mm)	0.79±0.04	0.87±0.06	0.95±0.05	0.92±0.06	0.96±0.07	0.89±0.07
LVPW;s (mm)	1.15±0.07	1.04±0.05	1.12±0.06	1.04±0.05	1.09±0.07	1.04±0.08
LVPW;d (mm)	0.80±0.07	0.74±0.03	0.82±0.06	0.80±0.03	0.84±0.04	0.88±0.07
MV E (mm/s)	640.31±37.99	724.69±20.07	590.68±27.73	554.54±41.94	601.29±32.32	690.80±22.21
MV A (mm/s)	443.42±33.85	538.04±29.09	402.82±24.06	402.55±45.02	387.59±40.12	557.05±37.16
E' (mm/s)	-26.26±1.80	-26.90±0.81	-28.49±1.69	-20.77±1.88	-21.21±5.72	-20.35±1.57
A' (mm/s)	-21.79±2.12	-24.16±1.69	-22.43±2.01	-19.97±1.54	-17.83±4.28	-19.95±2.26
MV E/E'	-24.91±1.37	-27.16±1.10	-21.14±1.20	-27.77±2.05	-18.01±4.72	-35.42±2.47
MV E/A	1.47±0.05	1.37±0.06	1.48±0.05	1.45±0.08	1.65±0.12	1.28±0.09
E'/A'	1.31±0.16	1.17±0.09	1.32±0.08	1.08±0.11	1.33±0.08	1.18±0.17
A'/E'	0.86±0.10	0.91±0.08	0.79±0.06	1.03±0.11	0.78±0.06	1.07±0.17

Table S7. Echo parameters of GsMTx4 injected MW mice

	WT	MW	MW + Vehicle	MW + GSMXT4
Heart Rate (bpm)	538.71±9.31	499.67±34.39	527.59±10.15	535.60±11.48
Diameter;s (mm)	2.19±0.08	2.58±0.12	2.53±0.03	2.17±0.08
Diameter;d (mm)	3.34±0.11	3.51±0.11	3.57±0.05	3.27±0.07
Volume;s (μL)	16.24±1.44	24.58±2.76	23.16±0.70	15.95±1.38
Volume;d (μL)	45.80±3.44	51.60±3.81	53.31±1.67	43.44±2.27
Stroke Volume (μL)	29.56±2.16	27.02±1.22	30.15±1.33	27.50±1.00
Ejection Fraction (%)	64.66±1.19	52.99±2.07	56.49±1.12	63.65±1.60
Fractional Shortening (%)	34.45±0.88	26.64±1.25	28.91±0.74	33.69±1.15
Cardiac Output (mL/min)	15.99±1.36	13.46±1.00	15.87±0.53	14.75±0.73
LV Mass (mg)	89.57±6.80	104.10±12.61	106.42±3.40	108.13±6.97
LV Mass Corrected (mg)	71.65±5.44	83.28±10.09	85.14±2.72	86.50±5.57
LVAW;s (mm)	1.10±0.06	1.08±0.09	1.19±0.04	1.32±0.06
LVAW;d (mm)	0.83±0.04	0.88±0.09	0.95±0.04	1.06±0.06
LVPW;s (mm)	1.14±0.07	1.08±0.07	1.07±0.02	1.18±0.08
LVPW;d (mm)	0.79±0.06	0.81±0.07	0.75±0.02	0.83±0.07
MV E (mm/s)	693.97±53.64	618.48±57.48	706.10±21.80	670.25±64.67
MV A (mm/s)	467.25±31.35	445.29±33.63	474.76±22.79	515.25±39.28
E' (mm/s)	-28.82±3.34	-19.23±1.38	-21.73±1.92	-26.07±2.65
A' (mm/s)	-27.31±1.98	-30.29±1.82	-28.15±2.04	-27.83±2.93
MV E/E'	-24.71±1.37	-32.11±2.16	-33.57±2.70	-26.27±2.65
MV E/A	1.49±0.08	1.42±0.16	1.50±0.05	1.29±0.06
E'/A'	1.11±0.18	0.66±0.08	0.78±0.07	0.99±0.14
A'/E'	1.03±0.16	1.64±0.20	1.33±0.12	1.11±0.15

Table S8. Echo parameters of Yoda1 injected WT mice

	WT	WT + Vehicle	WT + Yoda1
Heart Rate (bpm)	544.42±14.48	500.95±8.17	518.57±8.85
Diameter;s (mm)	2.24±0.09	2.21±0.08	2.60±0.07
Diameter;d (mm)	3.44±0.13	3.33±0.14	3.49±0.10
Volume;s (μL)	17.26±1.69	16.56±1.59	24.74±1.69
Volume;d (μL)	49.41±4.64	45.80±4.55	51.07±3.59
Stroke Volume (μL)	32.15±3.00	29.24±3.00	26.33±2.22
Ejection Fraction (%)	65.07±0.64	63.78±0.71	51.38±1.53
Fractional Shortening (%)	34.79±0.47	33.74±0.54	25.62±0.98
Cardiac Output (mL/min)	17.33±1.28	14.75±1.72	13.62±1.07
LV Mass (mg)	102.50±7.44	89.67±3.55	95.52±3.12
LV Mass Corrected (mg)	82.00±5.95	71.73±2.84	76.42±2.50
LVAW;s (mm)	1.20±0.10	1.11±0.05	1.05±0.05
LVAW;d (mm)	0.88±0.07	0.87±0.04	0.87±0.05
LVPW;s (mm)	1.20±0.07	1.15±0.02	0.99±0.04
LVPW;d (mm)	0.84±0.05	0.76±0.03	0.75±0.03
MV E (mm/s)	704.82±28.57	678.70±28.63	703.80±41.91
MV A (mm/s)	445.72±18.19	514.10±32.74	470.08±27.40
E' (mm/s)	-26.19±2.12	-25.02±2.62	-20.03±1.11
A' (mm/s)	-31.83±2.79	-32.58±4.30	-26.17±1.46
MV E/E'	-27.44±1.54	-27.97±1.80	-35.37±1.93
MV E/A	1.59±0.08	1.34±0.07	1.51±0.08
E'/A'	0.84±0.08	0.80±0.08	0.78±0.06
A'/E'	1.23±0.10	1.30±0.11	1.33±0.11

Table S9. Echo parameters of AAV9-Ctrl or AAV9-FOXO3/PPARγ/PPARα injected MW mice

	WT + AAV9-Ctrl	MW + AAV9-Ctrl	MW + AAV9-FOXO3	MW + AAV9-PPARγ	MW + AAV9-PPARα
Heart Rate (bpm)	500.93±26.94	495.09±23.59	535.53±17.42	486.02±44.39	498.09±20.81
Diameter;s (mm)	2.07±0.09	2.67±0.19	2.01±0.04	2.35±0.14	2.33±0.13
Diameter;d (mm)	3.09±0.13	3.56±0.18	3.02±0.06	3.46±0.19	3.39±0.18
Volume;s (µL)	14.24±1.53	27.38±4.72	13.03±0.61	19.67±2.70	19.27±2.58
Volume;d (µL)	38.25±3.69	54.22±6.82	35.77±1.73	50.58±6.41	48.17±6.08
Stroke Volume (µL)	24.01±2.23	26.84±2.36	22.73±1.24	30.90±3.76	28.89±3.51
Ejection Fraction (%)	62.90±1.03	50.89±2.97	63.50±0.91	61.45±0.97	60.19±0.37
Fractional Shortening (%)	33.00±0.74	25.47±1.78	33.49±0.69	32.14±0.59	31.23±0.14
Cardiac Output (mL/min)	11.98±1.21	13.31±1.42	12.16±0.70	14.35±1.25	14.27±1.54
LV Mass (mg)	84.39±3.49	117.20±16.56	92.33±9.02	110.37±11.99	109.10±10.24
LV Mass Corrected (mg)	67.51±2.79	93.76±13.25	73.86±7.21	88.30±9.59	87.28±8.19
LVAW;s (mm)	1.07±0.04	1.12±0.04	1.20±0.09	1.19±0.03	1.12±0.06
LVAW;d (mm)	0.90±0.03	0.99±0.04	0.94±0.06	1.01±0.04	0.97±0.07
LVPW;s (mm)	1.16±0.07	1.12±0.07	1.26±0.09	1.13±0.08	1.22±0.07
LVPW;d (mm)	0.80±0.07	0.80±0.06	0.89±0.10	0.78±0.06	0.86±0.07
MV E (mm/s)	652.20±33.87	763.79±29.62	595.15±36.80	699.83±32.10	699.64±60.63
MV A (mm/s)	404.04±32.56	541.75±47.36	507.18±19.63	593.61±57.86	481.06±50.21
E' (mm/s)	-24.08±1.42	-19.31±0.71	-22.20±1.98	-23.90±2.00	-24.19±2.09
A' (mm/s)	-21.48±1.85	-23.98±1.18	-19.50±1.18	-24.48±2.28	-20.93±1.60
MV E/E'	26.43±1.39	40.92±1.82	26.42±1.88	28.77±2.47	28.50±1.23
MV E/A	1.63±0.05	1.45±0.10	1.17±0.06	1.25±0.16	1.48±0.10
E'/A'	1.26±0.12	0.80±0.05	1.21±0.07	1.16±0.13	1.20±0.10
A'/E'	0.85±0.12	1.27±0.08	0.84±0.05	0.93±0.13	0.86±0.08

Table S10. Echo parameters of male WT and TG^{MyL2} mice

	WT (♂)	TG^{MyL2} (♂)
Heart Rate (bpm)	470.19±17.51	454.22±15.24
Diameter;s (mm)	2.01±0.08	2.45±0.13
Diameter;d (mm)	2.99±0.10	3.12±0.13
Volume;s (µL)	13.11±1.29	21.88±2.73
Volume;d (µL)	35.18±2.95	39.14±4.05
Stroke Volume (µL)	22.08±1.73	17.26±1.54
Ejection Fraction (%)	62.98±0.93	44.79±2.24
Fractional Shortening (%)	32.90±0.63	21.53±1.27
Cardiac Output (mL/min)	10.38±0.88	7.79±0.68
LV Mass (mg)	70.39±9.28	69.54±4.72
LV Mass Corrected (mg)	56.31±7.42	55.63±3.77
LVAW;s (mm)	0.94±0.06	0.84±0.06
LVAW;d (mm)	0.75±0.04	0.72±0.05
LVPW;s (mm)	1.05±0.07	0.94±0.05
LVPW;d (mm)	0.77±0.08	0.74±0.04
MV E (mm/s)	602.75±42.14	490.14±56.78
MV A (mm/s)	376.05±46.74	343.13±38.82
E' (mm/s)	-25.89±1.89	-17.17±1.59
A' (mm/s)	-20.12±1.37	-19.08±3.36
MV E/E'	-23.67±1.66	-28.22±1.53
MV E/A	1.70±0.17	1.45±0.09
E'/A'	1.33±0.13	0.98±0.10
A'/E'	0.81±0.10	1.08±0.11

Table S11. Echo parameters of female WT and TG^{MyL2} mice

	WT (♀)	TG^{MyL2} (♀)
Heart Rate (bpm)	537.08±11.29	535.96±24.80
Diameter;s (mm)	2.18±0.07	2.99±0.13
Diameter;d (mm)	3.22±0.08	3.56±0.10
Volume;s (μL)	16.07±1.29	35.51±3.72
Volume;d (μL)	42.07±2.46	53.55±3.78
Stroke Volume (μL)	25.99±1.26	18.04±0.93
Ejection Fraction (%)	62.09±1.06	34.64±2.59
Fractional Shortening (%)	32.48±0.70	16.19±1.34
Cardiac Output (mL/min)	13.88±0.51	9.67±0.66
LV Mass (mg)	76.18±4.56	82.40±2.36
LV Mass Corrected (mg)	60.95±3.64	65.92±1.89
LVAW;s (mm)	0.98±0.05	0.78±0.04
LVAW;d (mm)	0.75±0.04	0.70±0.03
LVPW;s (mm)	1.07±0.05	0.88±0.02
LVPW;d (mm)	0.75±0.04	0.72±0.02
MV E (mm/s)	605.57±22.12	704.10±40.84
MV A (mm/s)	407.79±33.95	556.02±44.51
E' (mm/s)	-21.41±0.89	-18.27±1.04
A' (mm/s)	-27.96±2.20	-24.82±2.27
MV E/E'	-28.46±1.09	-38.59±1.07
MV E/A	1.56±0.14	1.30±0.08
E'/A'	0.79±0.05	0.77±0.07
A'/E'	1.30±0.07	1.35±0.09

Table S12. Echo parameters of male WT and TG^{Myh6} mice

	WT (♂)	TG^{Myh6} (♂)
Heart Rate (bpm)	507.03±26.79	477.13±50.99
Diameter;s (mm)	2.03±0.08	3.12±0.10
Diameter;d (mm)	3.00±0.10	3.75±0.10
Volume;s (µL)	13.59±1.47	39.32±3.10
Volume;d (µL)	35.47±2.85	60.70±3.94
Stroke Volume (µL)	21.88±1.52	21.39±1.31
Ejection Fraction (%)	62.23±1.24	35.66±1.78
Fractional Shortening (%)	32.39±0.82	16.76±0.91
Cardiac Output (mL/min)	10.93±0.77	9.87±0.89
LV Mass (mg)	61.33±2.76	80.03±8.09
LV Mass Corrected (mg)	49.06±2.21	64.03±6.47
LVAW;s (mm)	0.98±0.03	0.77±0.05
LVAW;d (mm)	0.78±0.03	0.67±0.04
LVPW;s (mm)	0.89±0.05	0.73±0.05
LVPW;d (mm)	0.62±0.04	0.60±0.05
MV E (mm/s)	655.50±24.40	586.53±43.33
MV A (mm/s)	392.06±28.62	317.06±22.24
E' (mm/s)	-27.85±1.12	-17.26±1.38
A' (mm/s)	-20.98±0.73	-11.56±0.96
MV E/E'	-23.76±1.08	-34.42±1.57
MV E/A	1.72±0.09	1.86±0.08
E'/A'	1.34±0.06	1.51±0.08
A'/E'	0.76±0.03	0.68±0.03

Table S13. Echo parameters of female WT and TG^{Myh6} mice

	WT (♀)	TG^{Myh6} (♀)
Heart Rate (bpm)	480.17±21.55	431.88±60.33
Diameter;s (mm)	2.00±0.09	2.95±0.15
Diameter;d (mm)	2.95±0.10	3.48±0.13
Volume;s (μL)	13.01±1.46	34.39±4.49
Volume;d (μL)	34.05±2.74	50.71±4.62
Stroke Volume (μL)	21.03±1.40	16.31±2.34
Ejection Fraction (%)	62.27±1.62	32.60±4.72
Fractional Shortening (%)	32.43±1.07	15.33±2.53
Cardiac Output (mL/min)	10.05±0.71	7.27±1.56
LV Mass (mg)	69.77±4.84	69.35±4.44
LV Mass Corrected (mg)	55.82±3.87	55.48±3.55
LVAW;s (mm)	1.01±0.07	0.77±0.04
LVAW;d (mm)	0.82±0.08	0.67±0.04
LVPW;s (mm)	1.04±0.07	0.78±0.04
LVPW;d (mm)	0.75±0.07	0.62±0.04
MV E (mm/s)	561.50±49.55	632.45±64.05
MV A (mm/s)	355.96±40.96	280.49±30.51
E' (mm/s)	-24.86±2.22	-18.49±1.94
A' (mm/s)	-20.09±2.56	-17.27±1.63
MV E/E'	-22.88±1.65	-35.08±2.92
MV E/A	1.64±0.17	2.28±0.17
E'/A'	1.33±0.18	1.09±0.10
A'/E'	0.84±0.14	0.96±0.10

Table S14. Sequences of primers used in the study

Gene	Forward Primer (5'-3')	Reverse Primer (5'-3')
FtH	CCATCAACCGCCAGATCAAC	GAAACATCATCTCGGTCAAA
FtL	CGTCTCCTCGAGTTTCAGAAC	CTCCTGGGTTTTACCCCATTC
Fpn	GTGGAGTACTTCTTGCTCTGG	CTGCTTCAGTTCTGACTCCTC
TfR1	CTCAGTTTCCGCCATCTCAGT	GCAGCTCTTGAGATTGTTTGCA
Gapdh	ATCATCCCTGCATCCACT	ATCCACGACGGACACATT
Ptgs2	TGCTGGTGGAAAAACCTCGT	AAAACCCACTTCGCCTCCAA
Sod2	ACGTGAACAATCTCAACGCC	CCTTTGGGTTCTCCACCACC
Cpt1	AAGAACATCGTGAGTGGCGT	ACCTTGACCATAGCCATCCAG
Cpt2	CCCAAACCCAGTCGTGATGA	TGTGCCTGGATTTCTGAGGG
CD36	GATCGGAACTGTGGGCTCAT	ACTGGCATGAGAATGCCTCC
PGC-1α	ACTGGCATGAGAATGCCTCC	GGGCAATCCGTCTTCATCCA
PDK4	CACTGCTCCAACACCTGTGA	GCGTCTGTCCCATAACCTGA
Ucp3	GCAAAGTCCAACAGCCATCG	TCATCACGTTCCAAGCTCCC
Acsl1	ACCGTTGTAGAGTGTGTAATGC	GCAAAGTCCAACAGCCATCG
Gpam	ACAATGGCGAACAGTTGGGA	GGCTGTGCAAAATCCACTCG
Ech1	CCTGGCGGCTGATATTTCCA	GCTCTCGTCCACAGAATGGT

Table S15. Antibodies used for this study

**Antibodies**	**Source**	**IDENTIFIER/ Identifier**
4-HNE	Abcam	AB48506
PGC1-α	Abcam	AB313559
P-FOXO3A	Abcam	AB154786
Ferritin	Affinity	DF6278
CD36	Affinity	DF13262
CPT2	Affinity	DF7089
CPT1A	Affinity	DF12004
SLC40A1	Affinity	DF13561
GPX4	Affinity	DF6701
PDK4	Affinity	DF7169
P-PDH	Affinity	AF8502
PDH	Affinity	DF6680
CAMKII	Cell Signaling	4436
FOXO3A	Cell Signaling	12829
P-CAMKII	Cell Signaling	12716
β-Actin	Cell Signaling	4970
PIEZO1	Proteintech	15939-1-AP
SLC7A11	Proteintech	26864-1-AP
PPARα	Novus	NB300-537
PPARγ	Cell Signaling	2443

α-Actinin	Sigma-Aldrich	A7811
Peroxidase AfiniPure Goat Anti-Rabbit IgG(H+L)	Jackon	111-035-003
Peroxidase AffiniPure Goat Anti-Mouse IgG(H+L)	Jackon	115-035-003
Alexa Fluor® 594-conjugated AffiniPure Goat Anti-Mouse IgG (H+L)	Jackon	115-585-003
Alexa Fluor® 594-conjugated AffiniPure Goat Anti-Rabbit IgG (H+L)	Jackon	111-585-003
Alexa Fluor® 488-conjugated AffiniPure Goat Anti-Rabbit IgG (H+L)	Jackon	111-545-003
Alexa Fluor® 488-conjugated AffiniPure Goat Anti-Mouse IgG (H+L)	Jackon	115-545-003
DAPI	Abcam	AB104139

Movie S1. Molecular dynamics simulation of *WT* trimers.

Movie S2. Molecular dynamics simulation of *Piezo1*^{D674Y} mutant trimers.

Excel tables. Raw data used for quantification in the manuscript.

Full unedited gel for Supplemental Figure 3A

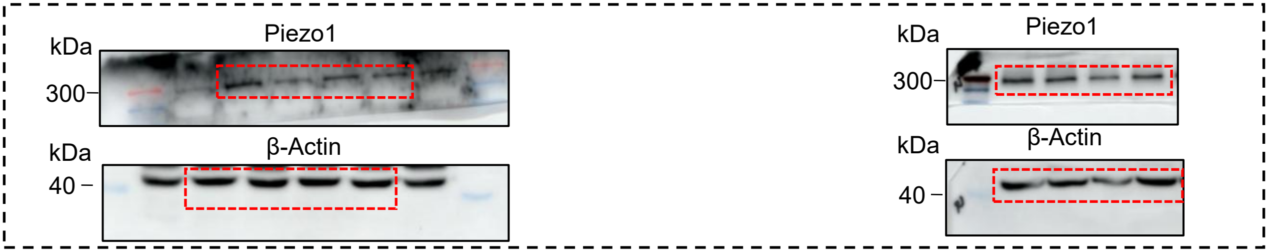

Full unedited gel for Supplemental Figure 5I

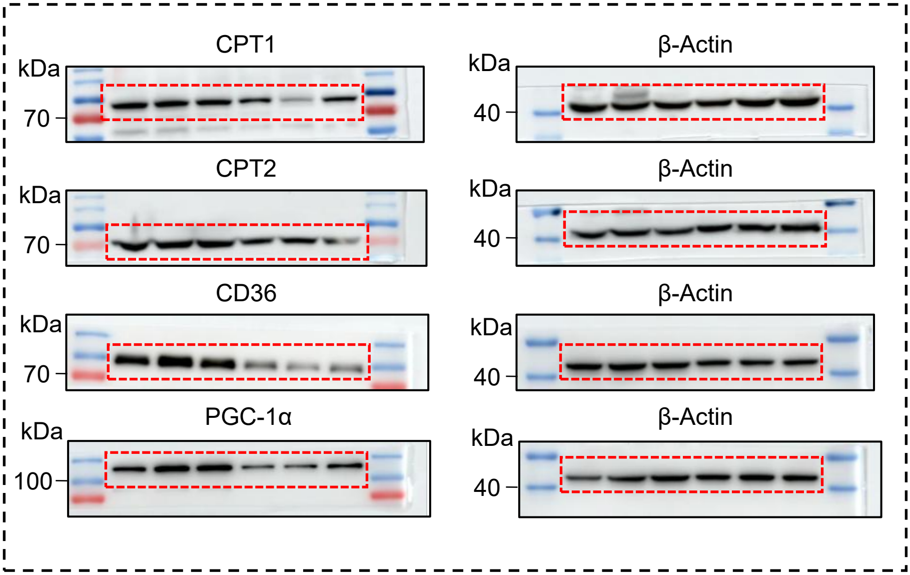

Full unedited gel for Supplemental Figure 5K

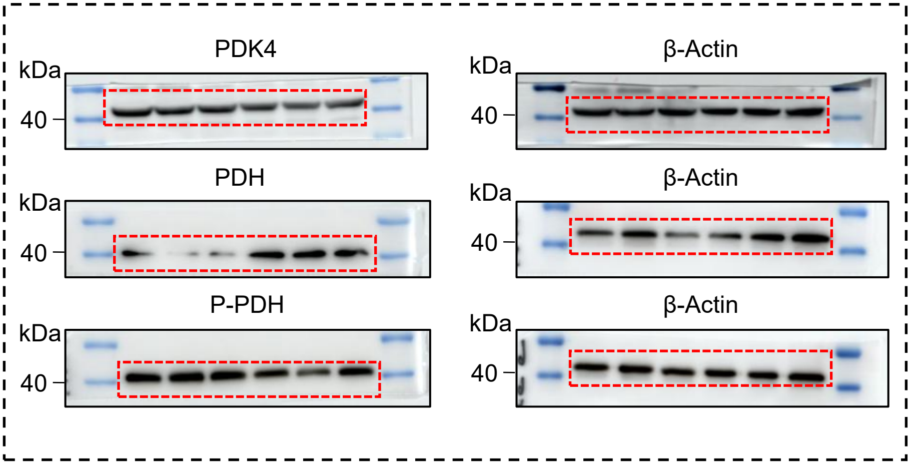

Full unedited gel for Supplemental Figure 6I

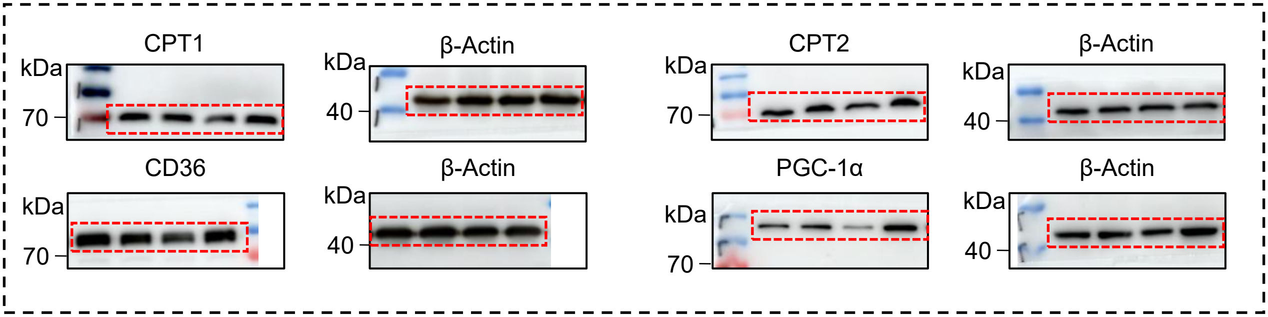

Full unedited gel for Supplemental Figure 6J

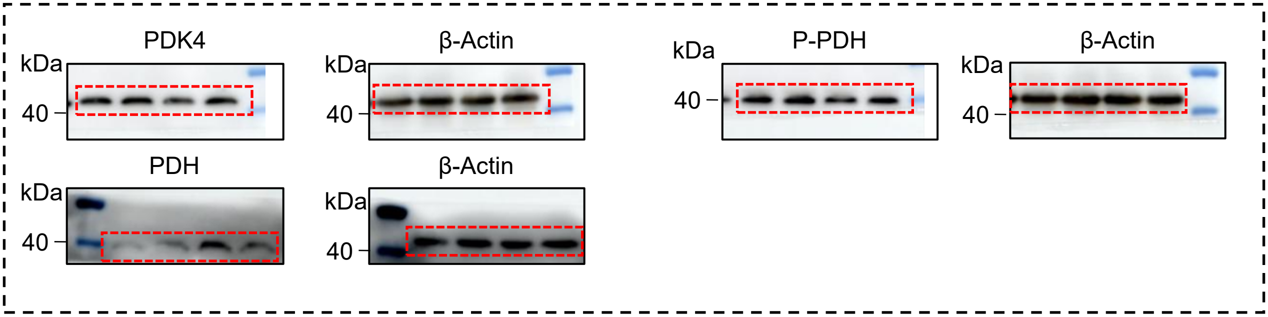

Full unedited gel for Supplemental Figure 7H

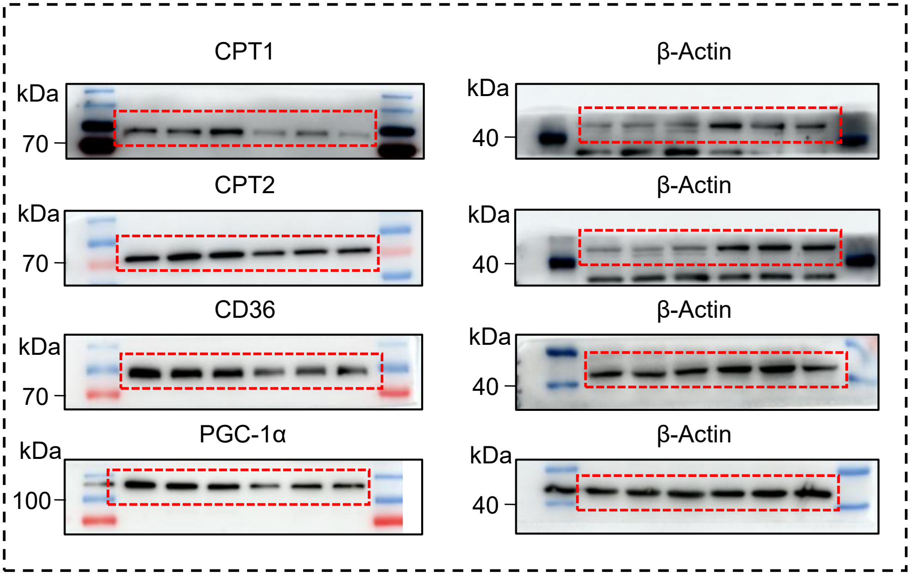

Full unedited gel for Supplemental Figure 7H

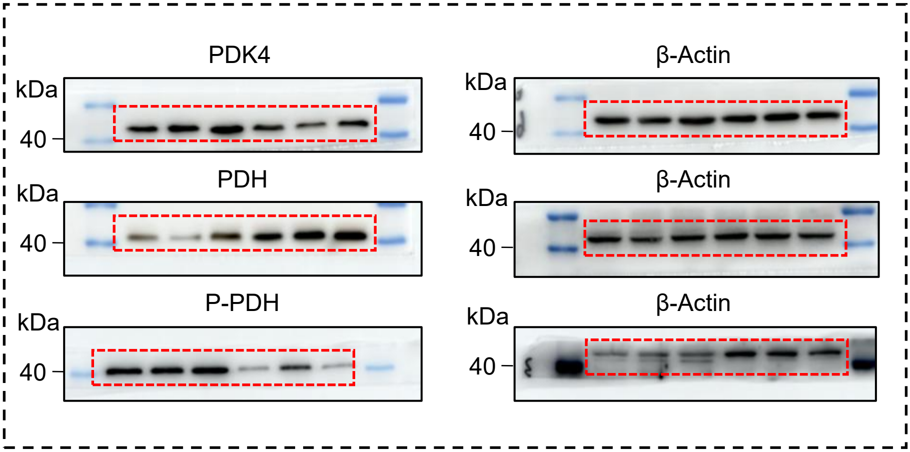

Full unedited gel for Supplemental Figure 7L

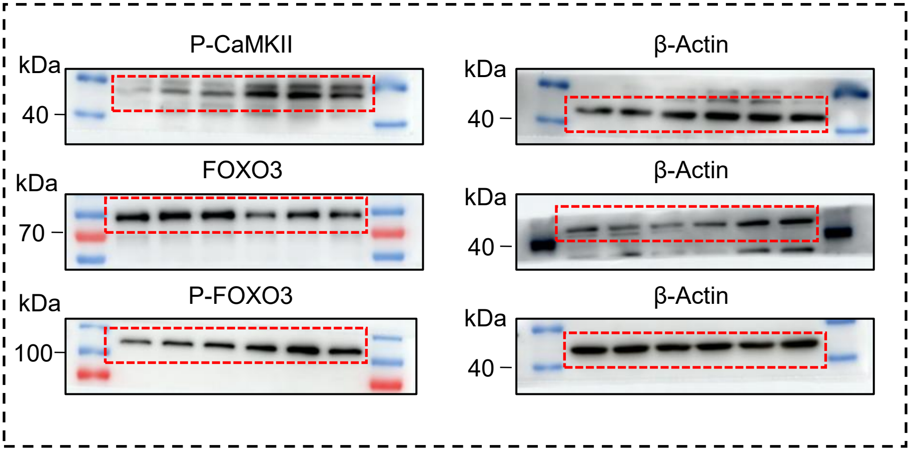

Full unedited gel for Supplemental Figure S7B

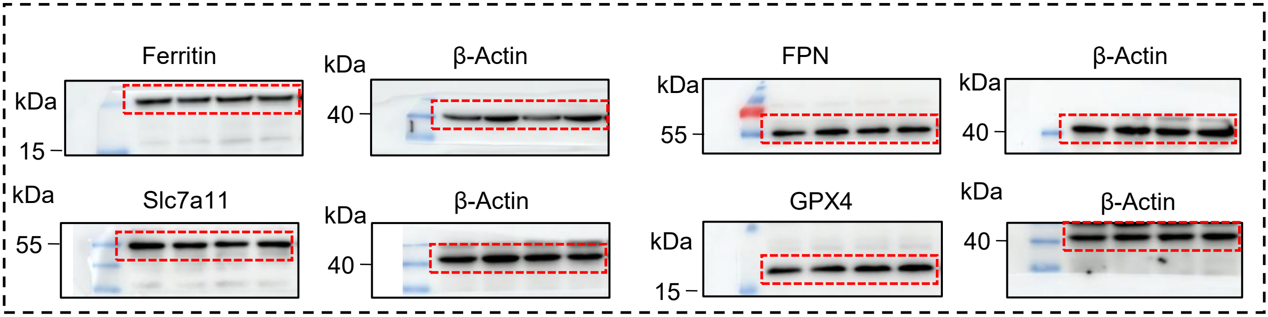

Full unedited gel for Supplemental Figure S11B

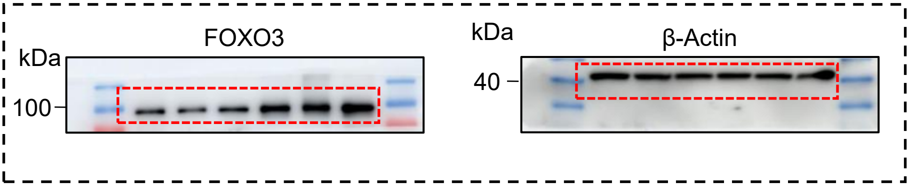

Full unedited gel for Supplemental Figure S11C

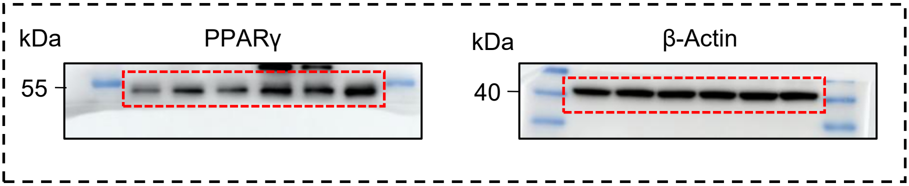

Full unedited gel for Supplemental Figure S11D

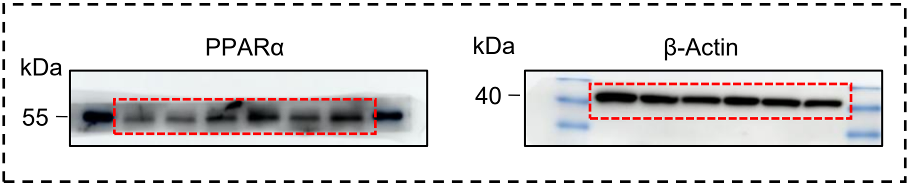

Full unedited gel for Supplemental Figure S12A

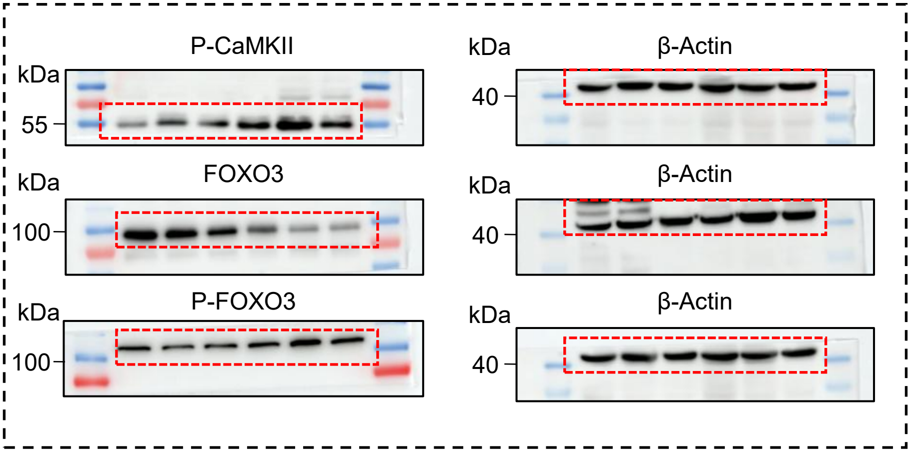

Full unedited gel for Supplemental Figure S17I

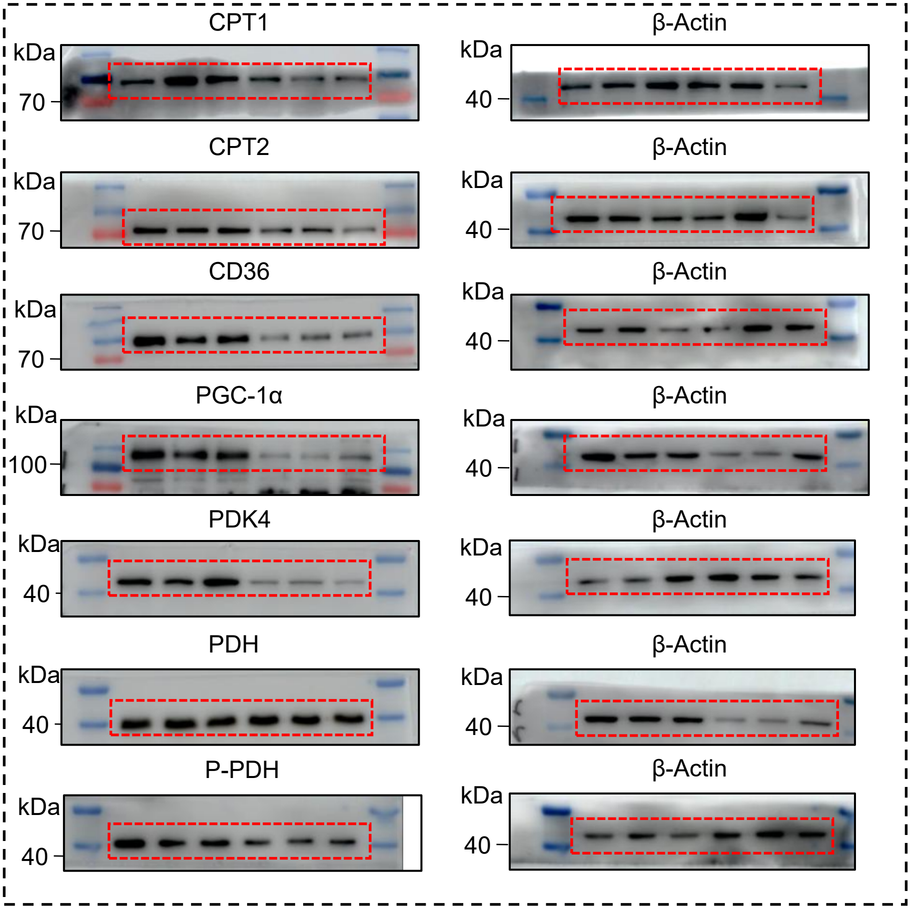

Full unedited gel for Supplemental Figure S17M

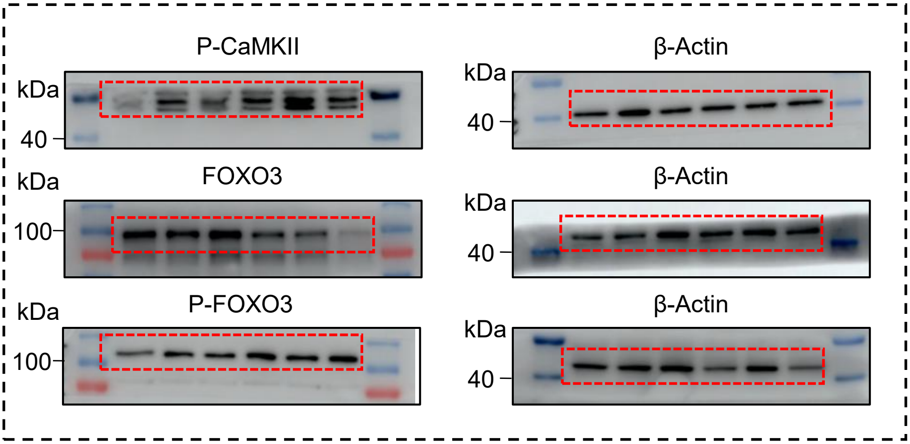

Supplement: Supplementary file 1 — Figs. S1 to S20 Tables S1 to S15 Legends for movies S1 and S2 Legend for dataset S1 Uncropped Western blots [file sciadv.ady9242_sm.pdf]
